# Characterization of contrasting rice (*Oryza sativa* L.) genotypes reveals the Pi-efficient schema for phosphate starvation tolerance

**DOI:** 10.1186/s12870-021-03015-4

**Published:** 2021-06-21

**Authors:** Suresh Kumar, Pallavi, Chetna Chugh, Karishma Seem, Santosh Kumar, K. K. Vinod, Trilochan Mohapatra

**Affiliations:** 1 Division of Biochemistry, ICAR-Indian Agricultural Research Institute, New Delhi , 110012 India; 2 Present Address: J.N.L. College, Patliputra University, Patna, Bihar India; 3 Decode Genomics Private Limited, New Delhi, India; 4 Division of Genetics, ICAR-Indian Agricultural Research Institute, New Delhi, India; 5 Indian Council of Agricultural Research, New Delhi, India

**Keywords:** Rice, Phosphorus starvation, Stress tolerance, Transcriptome analysis, Phosphatase, Transporter, Transcription factor, Root development

## Abstract

**Background:**

Phosphorus (P), being one of the essential components of nucleic acids, cell membranes and enzymes, indispensable for diverse cellular processes like photosynthesis/carbohydrate metabolism, energy production, redox homeostasis and signaling. Crop yield is severely affected due to Phosphate (Pi) deficiency; and to cope with Pi-deficiency, plants have evolved several strategies. Some rice genotypes are compatible with low Pi availability, whereas others are sensitive to Pi deficiency. However, the underlying molecular mechanism for low Pi tolerance remains largely unexplored.

**Result:**

Several studies were carried out to understand Pi-deficiency responses in rice at seedling stage, but few of them targeted molecular aspects/responses of Pi-starvation at the advanced stage of growth. To delineate the molecular mechanisms for low Pi tolerance, a pair of contrasting rice (*Oryza sativa* L.) genotypes [viz*.* Pusa-44 (Pi-deficiency sensitive) and its near isogenic line (NIL-23, Pi-deficiency tolerant) harboring *Phosphorus uptake 1* (*Pup1*) QTL from an *aus* landrace Kasalath] were used. Comparative morphological, physiological, and biochemical analyses confirmed some of the well-known findings. Transcriptome analysis of shoot and root tissues from 45-day-old rice plants grown hydroponically under P-sufficient (16 ppm Pi) or P-starved (0 ppm Pi) medium revealed that Pi-starvation stress causes global transcriptional reprogramming affecting several transcription factors, signaling pathways and other regulatory genes. We could identify several significantly up-regulated genes in roots of NIL-23 under Pi-starvation which might be responsible for the Pi starvation tolerance. Pathway enrichment analysis indicated significant role of certain phosphatases, transporters, transcription factors, carbohydrate metabolism, hormone-signaling, and epigenetic processes in improving P-starvation stress tolerance in NIL-23.

**Conclusion:**

We report the important candidate mechanisms for Pi acquisition/solubilization, recycling, remobilization/transport, sensing/signalling, genetic/epigenetic regulation, and cell wall structural changes to be responsible for P-starvation tolerance in NIL-23. The study provides some of the novel information useful for improving phosphorus-use efficiency in rice cultivars.

**Supplementary Information:**

The online version contains supplementary material available at 10.1186/s12870-021-03015-4.

## Background

Phosphorus (P) is one of the most important macronutrients necessary for the living organisms including plants. It is a vital constituent of several biological macromolecules like DNA, RNA and cell membrane, necessary for proper functioning of molecules like enzymes, ATP and NADPH etc., and essentially required for plant growth and development [1]. P is absorbed by roots mainly in the form of H_2_PO_4_^−^ or HPO_4_^−2^ from the soil. Though ample amount of P is present in soil, its availability in orthophosphate/inorganic phosphate (Pi) form is often hindered due to low solubility, immobility and inaccessibility of P to plants because of adsorption, precipitation, and/or conversion to organic form [2–4]. Pi-deficiency causes reduced growth of plant, curly leaves, hairy-lateral roots, purple pigmentation in leaves, and/or reduced tillering resulting in severe yield losses [5]. As per an estimate, 30 − 40% of the arable land world over has limited crop productivity mainly because of low Pi content [6]. In India, availability of Pi is low in upland soil, and it is worsening for most of the Indian soils [7]. In fact, Pi-deficiency in soil is becoming a global problem; hence, application of P-fertilizer in soil has become a necessity to ensure better productivity. This is the reason for a considerable increase in the global use of P-fertilizers in crop husbandry. On the contrary, P-use efficiency of crop plants has decreased to as low as < 20% [8]. However, continuous/extensive use of P-fertilizers would not be economically and ecologically sustainable because of the higher cost of P-fertilizers. Limited stocks of the rock phosphate [9], lower use-efficiency of applied P-fertilizers by crop plants, and excessive application of P-fertilizers lead to the environmental damage [10]. Therefore, the need of the day is to improve P-use efficiency of crop plants, and to explore the possibility of utilizing the naturally available P in the soil. While P acquisition refers to the Pi uptake through roots, P-use efficiency refers to the efficient remobilization/internal use of cellular Pi [11].

Length, number and branching of roots, lateral root/root-hair density and length (the root morphology), and root angle (root geometry), commonly represented as root system architecture (RSA), play important role in P-uptake from soil [12]. Plant modulates its RSA to increase root surface area for better P acquisition. Formation of root clusters has been observed in some plant species in response to low P in soil, which exudate organic acids to acidify/release the chelated ions around the roots, resulting in better availability of P and other micronutrients [13–15]. Plant releases phosphatase and RNase to enhance Pi availability and acquisition [16, 17]. Plant possesses specialized transporters and other molecular mechanisms for remobilizing Pi across the intracellular compartments where the P might have been stored in organic (phytic acid) form. Therefore, efforts are being made to comprehend the mechanisms involved in controlling Pi uptake/homeostasis in plants to improve P-use efficiency [18–20].

Attempts are also being made to analyze transcriptome data for different tissues from various plants, including rice, grown under different conditions until different developmental stages to identify the candidate genes/mechanisms associated with Pi-deficiency tolerance. However, most of the studies were carried out at the seedling stage [20–25]. Only a limited number of studies used different plant species at advanced stage of development [19–21]. Rice (*Oryza sativa* L.), one of the most important cereals, is a staple food for more than half of the global population [26]. An *aus* landrace of rice Kasalath was identified to be tolerant to P-deficiency, which led to the identification of a major quantitative trait loci (QTL) phosphorus uptake 1 (*Pup1*) mapped on the longer arm of chromosome 12 [27]. Previous efforts to link Kasalath allele at *Pup1* with known P-uptake related mechanisms showed that *Pup1* near-isogenic lines (NILs) had threefold higher P-uptake efficiency [28]. The *Pup1* QTL, generally absent in *Japonica* and *Indica* rice (modern) cultivars, has been reported to carry *Phosphate Starvation Tolerance 1* (*PSTOL1*) gene encoding a kinase to enhance Pi acquisition. However, the underlying mechanism for the functions of *Pup1* remains enigmatic [29, 30]. Although, no evident P-uptake gene was found to be located on *Pup1*, the QTL has a larger effect on P-deficiency tolerance [31]. Of the 68 gene models predicted on *Pup1*, most of them show sequence similarity with transposons, while others could not be annotated with confidence. Some of the genes code for putative fatty acid oxygenase, dirigent-like protein, aspartic proteinase, hypothetical proteins, and putative protein kinase [31].

Transcriptome analysis under P-starvation stress has been performed in many plants including Arabidopsis, rice, wheat, and maize [22, 24, 25, 32–37]. Most of the studies reported differential expression of genes mainly involved in P transport, phosphatases, transcription process, carbon metabolism/photosynthesis, lipid metabolism, cell wall remodeling, etc. Many of these transcriptome studies were conducted with short-duration P-stress only [20, 22, 25, 33–36]. In Arabidopsis, there are nine phosphorus transporter 1 (*PHT1*) family genes [38], and majority of the *PHT1* genes were reported to express in different parts of roots. In rice, 13 *PHT1* genes for high-affinity P transporters were reported to express [39, 40], some of which including *OsPHT1;1*, *OsPHT1;2*, *OsPHT1;3*, *OsPHT1;6*, and *OsPHT1;8* have been functionally characterized [19, 41–43]. A recent study showed that *OsPHT1;3* functions in extremely low-P environment to mediate Pi uptake, translocation and remobilization [19], while *OsPHT1;11* was reported earlier to be specifically activated by mycorrhizal symbiosis [40]. No significant induction of *OsPHT1;1* gene was reported in rice under very low Pi concentration [42]. Recently, a SULTR-like phosphorus distribution transporter (SPDT) was reported to play equally important role in distribution of Pi in rice [44].

To cope up with the P-deficiency stress, plants have evolved certain adaptive responses. The phosphorus transporters and related TFs like phosphorus starvation response (e.g. *PHR1*), SPX (a phosphate-dependent inhibitor of *PHR1*), etc. play crucial roles under P-deficiency stress. *OsPHR2* (involved in P-starvation signaling) positively regulates the expression of P-starvation inducible genes, like *OsPHTs*. Certain other genes have been reported to show variable expressed under P-starvation, which include high-affinity P transporter (*PHT1;6*) [41], *SPX* [45], and monogalactosyl diacylglycerol synthase (*MGD*) involved in galactolipid synthesis [46]. Moreover, the plant-specific WRKY family TFs have been reported to modulate transcription processes under abiotic stresses [47, 48]. The expression of *PHT1* genes was reported to be regulated by TFs due to the presence of cis-acting elements in promoter [49, 50]. WRKY along with C2H2 zinc-finger domain containing TFs regulate transcription of target genes [51]. MYB2 functions as transcriptional activator of ABA-dependent/ABA-independent genes under abiotic stresses, and it also activates transcription of miR-399f in Arabidopsis under P-starvation [52]. miR-399 and miR-827 have been reported to positively regulate expression of the genes involved in phosphorus transport and utilization [53].

Phytohormones like auxin, cytokinin, ethylene, and ABA are involved in transcriptional regulation of the genes for P-starvation responses [54]. ABA-signaling was reported to affect root development, root architecture, root hair density, and root − shoot biomass ratio [55]. Optimum level of gibberellic acid (GA) was reported to be necessary for root-hair growth under P-deficiency. The stress reduces bioactive GA level, which causes accumulation of DELLAs and triggers the responses like alteration in root architecture, reduced shoot growth, and accumulation of anthocyanin in Arabidopsis [56]. P-starvation was reported to up-regulate GA biosynthetic gene *GA3-ox2* in *myb1* rice mutants, resulting in enhanced GA level and increased length of lateral roots [18]. This demonstrates that MYB1 mediates cross-talk between nutrient signaling and phytohormone signaling pathways. Studies have also reported enhanced expression of jasmonic acid biosynthetic and signaling genes in Arabidopsis and Sorghum [25, 57].

In the present study, comparative transcriptome analysis of shoot and root tissues from 45-day-old plants (at vegetative/tillering stage) of contrasting rice genotypes Pusa-44 (P-starvation sensitive) and NIL-23 (P-starvation tolerant) grown hydroponically under P-sufficient or P-starvation condition revealed some of the candidate genes/mechanisms involved in the stress tolerance. To cope up with Pi-deficiency, plants have evolved a number of strategies including morphological changes like increased length and density of lateral roots, formation of denser and longer root-hairs resulting in better foraging of soil. Biochemical changes like increased production of acid phosphatases and exudation of organic acids, molecular changes like dynamic regulation of gene expression, metabolic changes like reprogramming of carbohydrate and lipid metabolism to help improving P-use efficiency of the plant. Thus, the study provides insights into the differentially expressed genes involved in Pi transport, signaling, phosphatase synthesis, coding for TFs, core-histone domain containing proteins, and glycine-rich cell wall structural proteins to improve P-starvation tolerance in rice. The identified genes/mechanisms for P-starvation tolerance might be utilized in breeding programs to improve yielding potential of rice in P-deficient soils.

## Results

### Morphological and developmental changes in plants under P-starvation

**Fig. 1:**
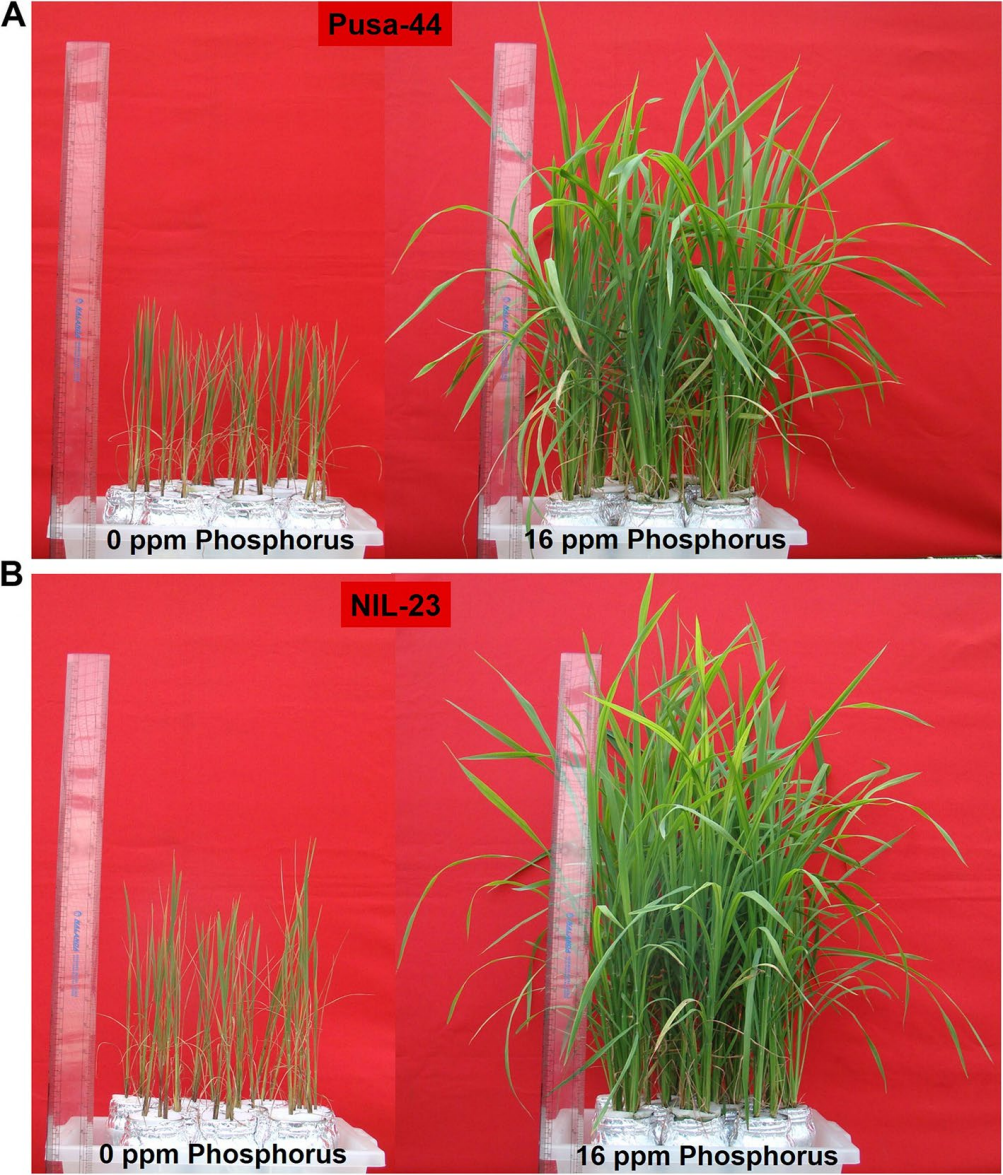
Shoot morphology of the contrasting rice genotypes. **A** Pusa-44, stress-sensitive recurrent parent, **B** near isogenic line (NIL)-23, stress-tolerant grown under Pi-starvation stress. Plants were grown until 45-days under control (16 ppm inorganic phosphorus) or treatment (0 ppm Pi) for comparative assessment

**Fig. 2:**
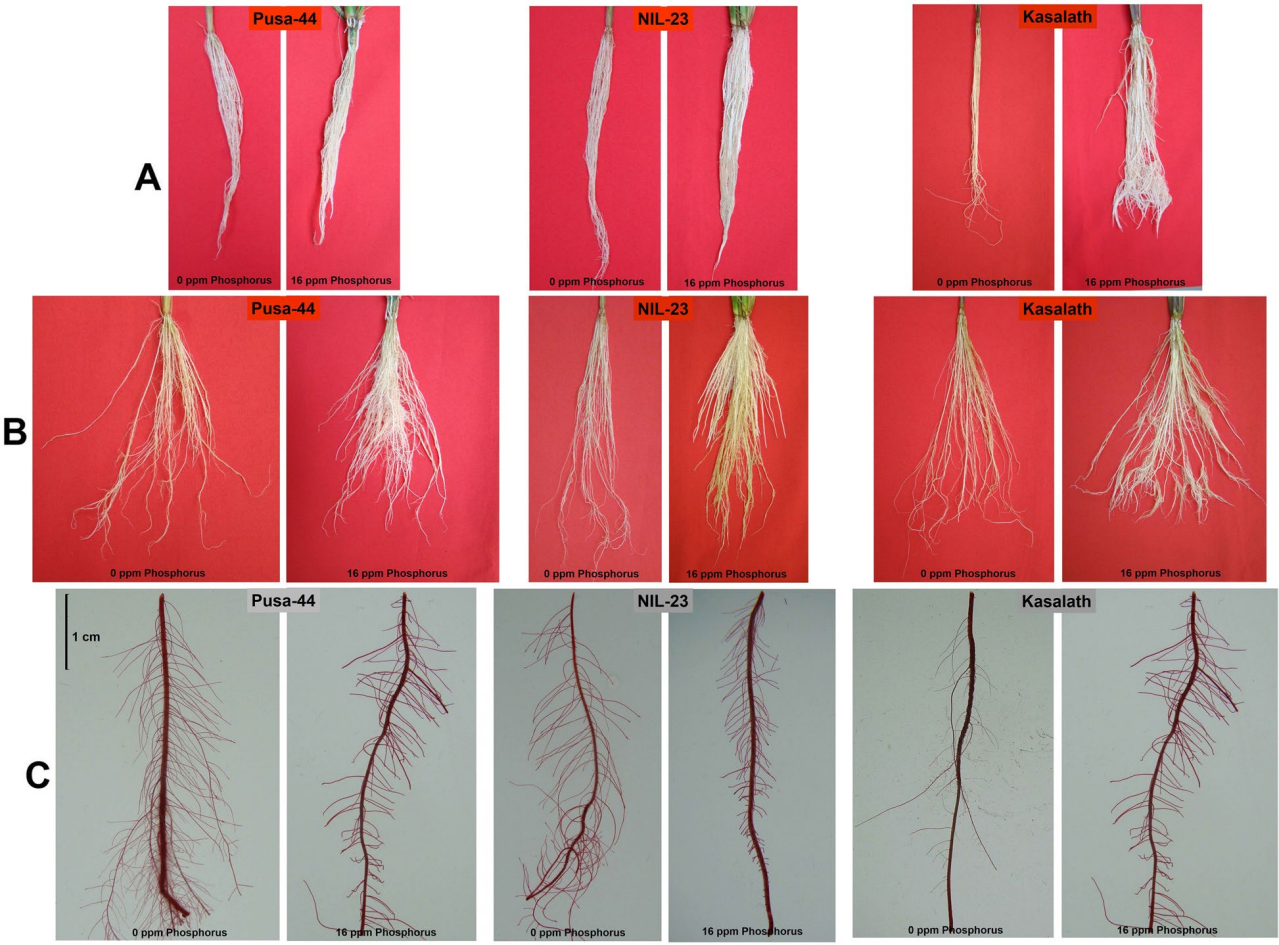
Root morphology of the contrasting rice genotypes (Pusa-44, stress-sensitive; NIL-23, stress-tolerant) under Pi-starvation stress compared with that of the *Pup1* QTL donor (Kasalath). **A** Comparision of the root bulkiness, **B** comparision of thickness and spread of the roots, **C** comparision of the secondary and tertiary roots. Roots of 45-day-old plants grown under control (16 ppm inorganic phosphorus) or treatment (0 ppm Pi) were used for comparative assessment

The contrasting rice (Pusa-44 and NIL-23) genotypes were grown hydroponically in the medium supplemented with/without Pi till the vegetative/tillering (45 days) stage. We observed significant changes in growth of roots due to P-starvation even at the seedling stage (20-day-old) of plant (Supplementary Fig. S1). Comparison of shoot morphology of the plants grown in hydroponics medium containing varying (0 to 40 ppm) concentration of Pi showed no obvious effects of higher (20 − 40 ppm) Pi in the medium. However, lower Pi concentration (0 − 12 ppm) in the medium significantly reduced shoot growth compared to the optimum (16 ppm) Pi content (Supplementary Fig. S2). Due to P-starvation stress, a considerable reduction in the growth of shoot and root was observed at vegetative (45 days old) stage of plant (Supplementary Fig. S3). A considerable effect of P-starvation stress was observed on height/biomass of the plants (Fig. 1, Supplementary Table S1). P-starvation/deficiency caused stunted growth, reduced tillering, and leaves were shorter and erect (Supplementary Fig. S4). At the vegetative stage, a significant reduction in growth of roots, in terms of the number and spread, due to P-starvation under hydroponic conditions was observed (Fig. 2A, B). Moreover, a significant increase in the size of tertiary root/root-hairs was observed due to P-starvation stress in the rice genotypes (Fig. 2C).

### Effect on root − shoot biomass ratio

Comparative analysis of root and shoot biomass produced by the contrasting rice genotypes under P-starvation stress indicated that the stress has considerable effects on the biomass production. However, the effect of stress was more prominent on shoot biomass production compared to that in root. A considerable reduction (63%) in shoot biomass was observed due to P-starvation stress. More importantly, the reduction in root biomass was comparatively lesser (50% in P-deficiemcy sensitive, and 36 − 41% inP-deficiency tolerant genotype) compard to that in shoot. Moreover, root − shoot biomass ratio incrased significantly under the stress in both the stress-sensitive and stress-tolerant rice genotypes (Supplementary Table S1). The root − shoot biomass ratio was observed to be higher (0.5) in case of NIL-23 (stress-tolerant), compared to that (0.286) in Pusa-44 (stress-sensitive) genotype.

### Effect of P-starvation on leaf morphology and chlorophyll content

A considerable decrease in size and area of the leaf was observed under P-starvation stress in the rice genotypes. More than 50% reduction in length and 72% reduction in width of the leaf was observed in case of P-deficiency sensitive genotype (Pusa-44), while 41 − 44% reduction in length and 54 − 60% reduction in width was observed in the P-deficiency tolerant (NIL-23 and Kasalath) rice genotypes (Supplementary Fig. S5). Total chlorophyll content in leaf decreased significantly with decreasing availability of Pi in the hydroponic medium. However, the decrease was considerably higher when Pi was absent from the medium. The response to P-starvation stress was more prominent in Pusa-44 (P-deficiency sensitive) compared to that observed in NIL-23 (P-deficiency tolerant) genotype (Supplementary Fig. S6).

### Changes in intrinsic and secreted acid phosphatase activity

APase activity increased significantly in roots compared to that observed in shoot under P-starvation stress. The increase in APase activity was more prominent in root of the P-deficiency tolerant rice genotypes. With increasing deficiency of phosphorus, the APase activity increased significantly, particularly in the P-deficiency tolerant genotypes. APase activity was also observed to increase in shoot with increasing deficiency of phosphorus in the hydroponic medium (Supplementary Fig. S7).

Secretion of APase from roots was obserbed to increase under P-deficiency stress in all the three rice genotypes. With the increasing deficiency of P in the hydroponic medium, secretion of APases increased significantly, particularly in P-tolerant rice genotype. However, comparatively lesser secretion of APase was recored from roots when the plants were grown under P-satrvation stress (Supplementary Fig. S8). A significant decrease in pH of the medium was recorded in presence of Pi in the medium. The decrease in pH of the medium was recorded to be more with increasing comcentartion of Pi in the medium (Supplementary Table S2).

### Mobilization of phosphorus in plant tissues

A significant reduction in P content was observed with increasing deficiency of Pi in the hydropnic mdium. Most of the phosphorus acquired by roots is mobilized to shoots through P transporters, partcularly in case of the P-deficiency tolerant genotypes. Therefore, phosphorus content of shoot was observed to be higher (1.6 − 2.3-fold) than that of the root (Supplementary Fig. S9). The ability of P-deficiency tolerant (NIL-23 and Kasalath) genotypes to acquire and accumulate P was observed to be better. Similarly, mobilization of phosphorus from root to shoot was observed to be better, particularly in case of the tolerant genotypes.

### Transcriptome library preparartion, sequencing and mapping on reference genome

To have comprehensive understanding of the mechanisms involved in Pi-starvation stress tolerance in rice, contrasting rice genotypes for responses to P-deficiency stress were used. A total of 16 libraries for root and shoot tissues from two rice genotypes grown until tillering stage under Pi-sufficient or –deficient conditions were successfully prepared in two replications for whole transcriptome analysis. Total of 395 million reads with an average of 25 million reads for each sample were generated. Reference-based mapping of RNA-seq data on rice reference genome (TIGR v7) using HiSat2 and Stringtie showed ~ 87% uniquely mapped reads (Table 1).

**Table 1:** Summary of transcriptome data mapping statistics

**Sample ID**	**Replication**	**Description**^**a**^	**Total reads**	**Trimmed reads **	**Mapping efficiency (%)**
PSF_R1	1	Pusa-44, Shoot, Full (16 ppm) Pi	23,920,000	22,571,192	95.07%
PSF_R2	2		22,765,118	21,575,530	95.02%
PSZ_R1	1	Pusa-44, Shoot, Zero (0 ppm) Pi	28,000,800	25,864,646	94.04%
PSZ_R2	2		27,064,264	25,220,216	94.11%
PRF_R1	1	Pusa-44, Root, Full (16 ppm) Pi	24,600,000	22,326,862	90.62%
PRF_R2	2		24,349,572	22,284,610	90.51%
PRZ_R1	1	Pusa-44, Root, Zero (0 ppm) Pi	20,400,000	17,204,480	65.70%
PRZ_R2	2		20,087,780	16,990,208	65.48%
NSF_R1	1	NIL-23, Shoot, Full (16 ppm) Pi	24,200,000	22,484,456	94.03%
NSF_R2	2		23,828,102	22,263,540	93.93%
NSZ_R1	1	NIL-23, Shoot, Zero (0 ppm) Pi	28,048,000	26,021,404	89.52%
NSZ_R2	2		28,000,318	26,133,560	89.37%
NRF_R1	1	NIL-23, Root, Full (16 ppm) Pi	26,300,000	24,010,636	88.02%
NRF_R2	2		26,105,496	24,097,160	88.21%
NRZ_R1	1	NIL-23, Root, Zero (0 ppm) Pi	23,800,000	22,380,692	79.68%
NRZ_R2	2		23,614,190	22,281,334	79.35%

^a^The rice plants were grown hydroponically in PusaRicH medium containing full (16 ppm) inorganic phosphorus (Pi) or no/zero (0 ppm) Pi in the medium

### Differentially expressed genes in contrasting rice genotypes

**Fig. 3:**
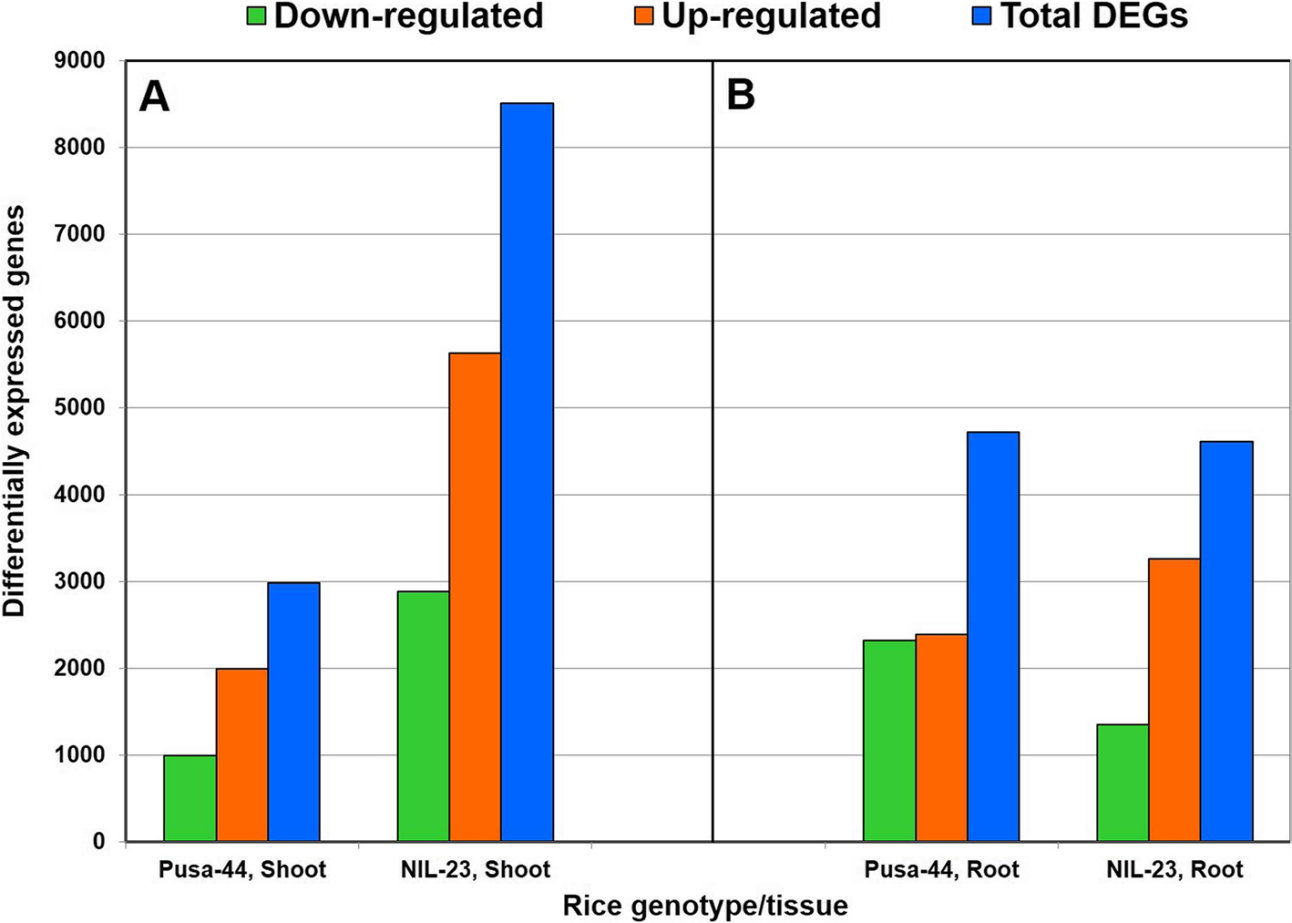
Differentially expressed genes (DEGs) under P-starvation stress in the stress-sensitive (Pusa-44) and stress-tolerant (NIL-23) rice genotypes grown hydroponically. Shoot and root tissues were collected from 45-day-old plants, continuously grown under control (16 ppm inorganic phosphorus) or treatment (0 ppm Pi) and used for whole transcriptome analysis

To decipher the genes/mechanisms involved in P-starvation stress tolerance in rice, comparative analysis of transcriptome data for root and shoot was performed which resulted in the identification of differentially expressed genes (DEGs) up- or down-regulated based on log_2_-FC (fold change) and false discovery rate (FDR) p < 0.05. Since the contrasting rice genotypes grown hydroponically in the medium containing 0 or 16 ppm Pi were used in the present study, four comparison groups were made for the analysis of DEGs involved in P-starvation tolerance: (i) Roots from Pusa-44 (stress-sensitive genotype) grown in full (16 ppm Pi, control) vs zero (0 ppm Pi, treated). This resulted in identification of 4716 DEGs with 2393 up-regulated and 2323 down-regulated genes under the stress. (ii) Roots from NIL-23 (stress-tolerant genotype) grown in full Pi (control) vs zero Pi (treated), which resulted in the identification of 4611 DEGs, with 3259 up-regulated and 1352 down-regulated genes under the stress. (iii) Shoots from Pusa-44 grown in full vs zero Pi, showing 2985 DEGs comprising of 1992 up-regulated and 993 down-regulated genes. Similarly, (iv) the comparison of shoots from NIL-23 grown in full vs zero revealed 8515 DEGs including 5627 up-regulated and 2888 down-regulated genes under the P-starvation stress (Fig. 3).

**Fig. 4:**
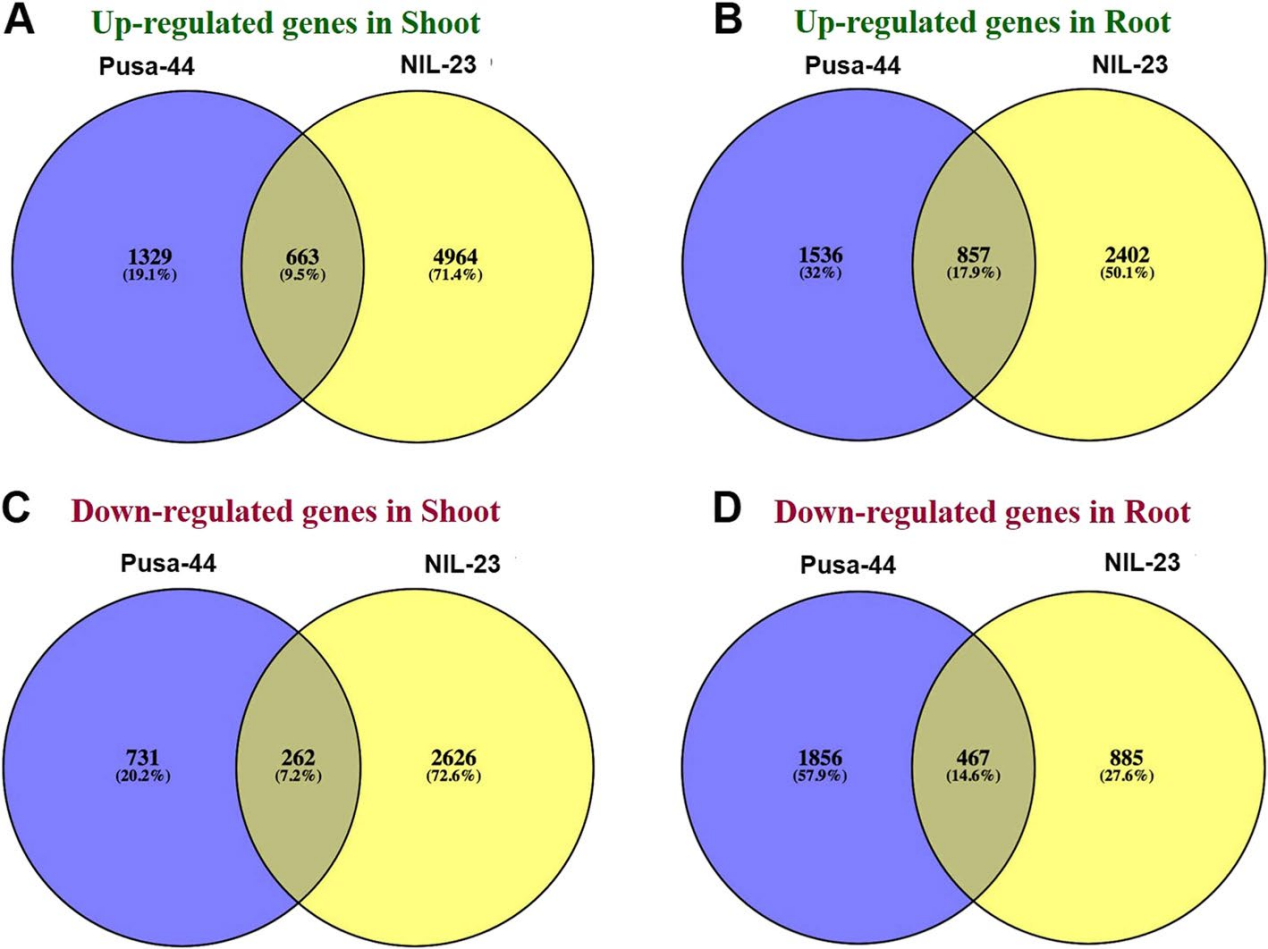
Differentially expressed genes (DEGs) in stress-sensitive (Pusa-44) and stress-tolerant (NIL-23) rice genotypes grown hydroponically under P-starvation stress. **A** Differentially and commonly up-regulated genes in shoot of the contrasting rice genotypes, **B** differentially and commonly up-regulated genes in root of the contrasting rice genotypes, **C** differentially and commonly down-regulated genes in shoot of the contrasting rice genotypes, **D** differentially and commonly down-regulated genes in root of the contrasting rice genotypes. Shoot and root tissues were collected from 45-day-old plants, continuously grown under control (16 ppm inorganic phosphorus) or treatment (0 ppm), and used for whole transcriptome analysis

In roots of the stress-tolerant rice genotype (NIL-23), 2402 (50.1% of the DEGs) genes were observed to be exclusively up-regulated, compared to only 1536 (32%) genes up-regulated in stress-sensitive genotype (Pusa-44), along with 857 (17.9%) commonly up-regulated genes in the contrasting rice genotypes under the stress (Fig. 4A). Among the down-regulated genes, 1856 were observed to be down-regulated in roots of stress-sensitive genotype (Pusa-44) compared to only 885 genes down-regulated in Pusa-44, with 467 (14.6%) genes commonly down-regulated in both the genotypes under the stress (Fig. 4B). However, 262 (7.2%) genes were commonly down-regulated in shoots of the contrasting rice genotypes due to P-starvation stress.

Similarly, in shoots of the contrasting rice genotypes, 4964 (71.4%) genes were observed to be exclusively up-regulated in the stress-tolerant rice genotype compared to only 1329 (19.1%) genes up-regulated in stress-sensitive genotype, with 663 (9.5%) genes commonly up-regulated in both the genotypes under the stress (Fig. 4C). Among the down-regulated genes in shoots, 2626 (72.6%) genes were observed to be down-regulated in root of the stress-sensitive genotype compared to only 731 (20.2%) genes down-regulated in the stress-sensitive genotype, with 262 (7.2%) genes commonly down-regulated in both the genotypes under the stress (Fig. 4D). Remarkably, the maximum number of DEGs (50 − 71%) were observed to be up-regulated in shoot and root of the stress-tolerant (NIL-23) genotype under P-starvation stress.

**Fig. 5:**
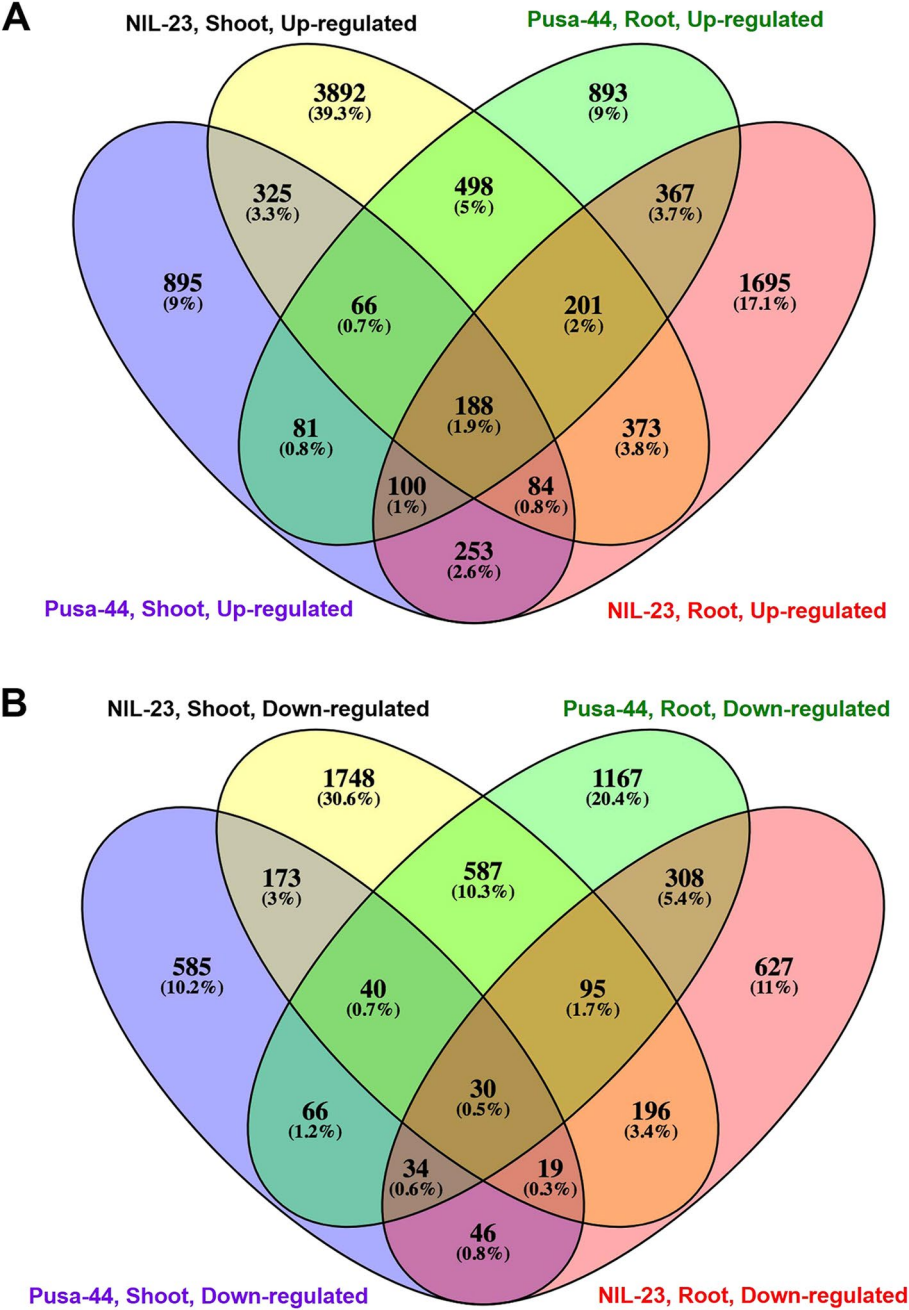
Four-way analysis of the differentially expressed genes (DEGs) in stress-sensitive (Pusa-44) and stress-tolerant (NIL-23) rice genotypes grown hydroponically under P-starvation stress. **A** Genotype- and tissue-wise differentially and commonly up-regulated genes in the contrasting rice genotypes, **B** genotype- and tissue-wise differentially and commonly down-regulated genes in the contrasting rice genotypes. Shoot and root tissues were collected from 45-day-old plants, continuously grown under control (16 ppm inorganic phosphorus) or treatment (0 ppm), and used for whole transcriptome analysis

Up-regulated expression of the genes played significant role in stress tolerance, particularly in roots. While 1695 (17.1%) genes were exclusively up-regulated in root of NIL-23, it was only 893 (9%) in root of Pusa-44. Similarly, 3892 (39.3%) genes were exclusively up-regulated in shoot of NIL-23, it was only 895 (9%) in shoot of Pusa-44. Moreover, only 367 (3.7%) and 325 (3.3%) genes were commonly up-regulated in roots and shoots of both the genotypes, respectively (Fig. 5A).

Down-regulation of the genes played important role in managing stress tolerance in shoot of NIL-23. While 1748 (30.6%) genes were exclusively down-regulated in shoot of NIL-23, it was only 585 (10.2%) in shoot of Pusa-44. Similarly, 627 (11%) genes were exclusively down-regulated in root of NIL-23, it was only 4467 (20.4%) in root of Pusa-44. Moreover, only 173 (3%) and 308 (5.4%) genes were commonly down-regulated in shoot and root of both the genotypes, respectively (Fig. 5B).

### Differential expression of the key phosphorus-responsive genes

**Fig. 6:**
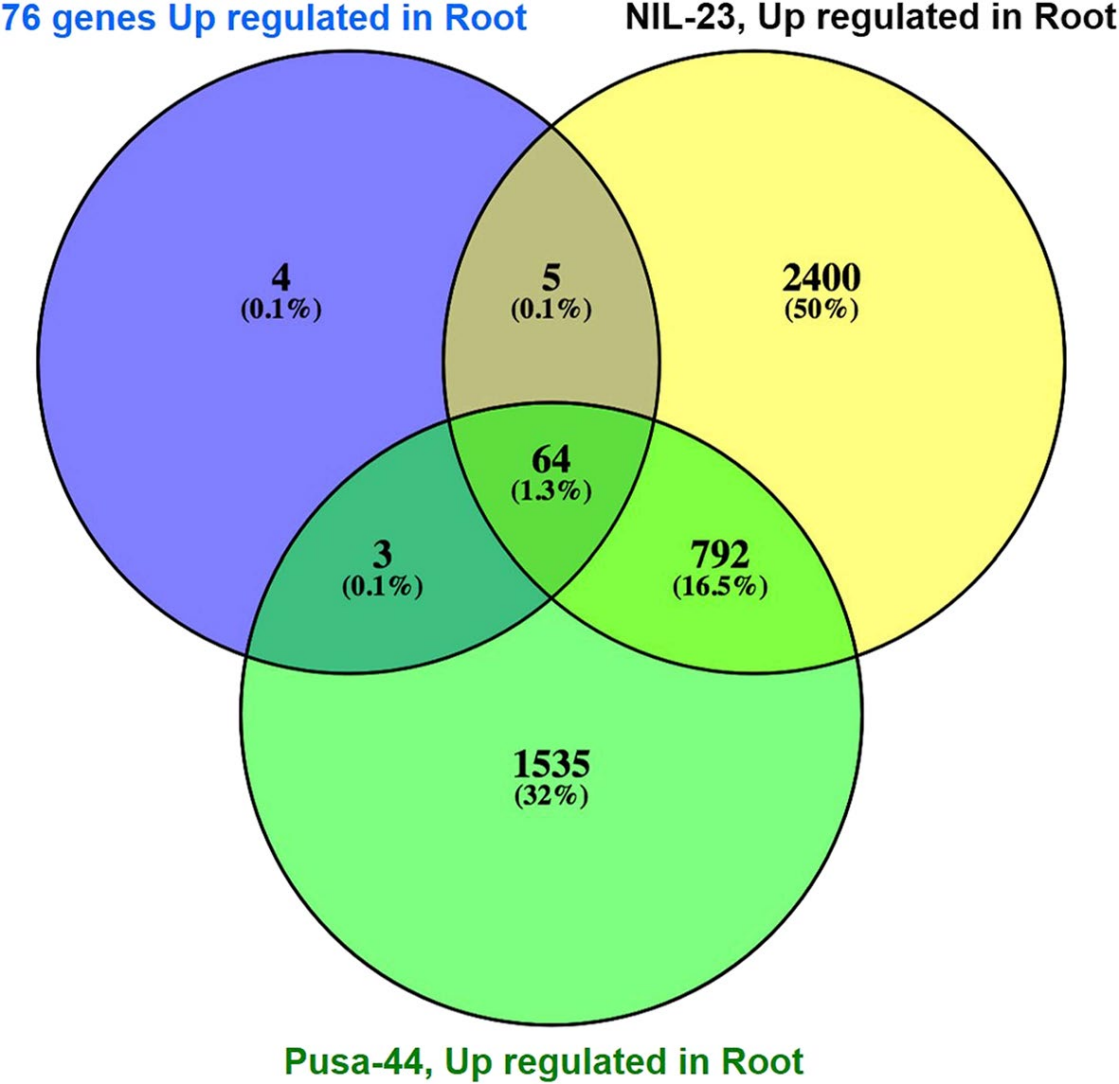
Expression of the known (76_up_genes_root) Pi-starvation–inducible genes in the stress-sensitive (Pusa-44) and stress-tolerant (NIL-23) rice genotypes grown hydroponically under P-starvation stress. Shoot and root tissues were collected from 45-day-old plants, continuously grown under control (16 ppm inorganic phosphorus) or treatment (0 ppm Pi), and used for whole transcriptome analysis

To assess the imposition of P-starvation stress, we tested the expression level of certain P-starvation-inducible genes reported earlier. Our transcriptome data indicated that in root of the NIL-23 69 P-starvation-inducible genes were up-regulated, whereas in root of the stress-sensitive (Pusa-44) genotype 67 genes were up-regulated (Supplementary Table S3). Some of the known Pi-starvation–inducible genes, namely phosphoethanolamine/phosphocholine phosphatase (LOC_Os01g52230), purple acid phosphatase (LOC_Os08g17784), glycosyl transferase group 1 domain containing protein (LOC_Os01g04920), phosphoesterase family protein (LOC_Os11g38050), SPX2 domain-containing protein (LOC_Os03g29250), Ser/Thr protein phosphatase family protein (LOC_Os07g01540), and inorganic phosphate transporter (LOC_Os06g21950), showed > eightfold up-regulated expression in root of NIL-23 under the stress. Out of the 76 known Pi-starvation–inducible genes [33], 5 of the genes were expressed in root of NIL-23 only, while 64 genes were commonly expressed in both the genotypes (Fig. 6).

Other genes like soluble inorganic pyrophosphatase (LOC_Os05g02310) and glycosyl hydrolases family 16 (LOC_Os06g48200) showed considerable up-regulation in root of NIL-23 under the stress. Moreover, six genes for Ser/Thr protein phosphatase family protein (LOC_Os07g01540, LOC_Os11g05400, LOC_Os12g44020, LOC_Os11g34710, LOC_Os03g13540, and LOC_Os07g04210) were observed to be up-regulated in root of NIL-23 during the stress (Supplementary Table S3). Moreover, we observed > 6.3-fold and > 2.4-fold up-regulated expression of LOC_Os08g33710 and LOC_Os01g67190 genes, respectively, for ribonuclease T2 family domain containing proteins in roots of NIL-23 under the stress.

### Dynamism in expression of P-transporter during P-starvation

Expressions of Phosphorus-transporters in rice (*OsPHT*s) showed dynamic variation among the genotypes and tissues. Expressions of six *PHT1* genes (*OsPHT1;6*, *OsPHT1;10; OsPHT1;4*, *OsPHT1;5*, *OsPHT1;2*, *OsPHT1;8*) were observed to be highly up-regulated and in roots of NIL-23 under P-starvation, whereas *OsPHT1;7* showed down-regulated expression (Supplementary Table S4). Interestingly, the fold change in expression of *OsPHTs* in roots of NIL-23 was more compared to that in root of Pusa-44. *OsPHT1;6* showed the highest (12.65-fold) level of expression in roots of NIL-23. More importantly, *OsPHT1;9* showed exclusive expression (3.52-fold up-regulated) in root of NIL-23. Moreover, *OsPHT1;12* expression was observed to be significantly more up-regulated in shoots of the rice genotypes compared to that in roots (Supplementary Table S4), suggesting its role in mobilization of Pi from root to shoot.

### Differential expression of transcription factors

**Fig. 7:**
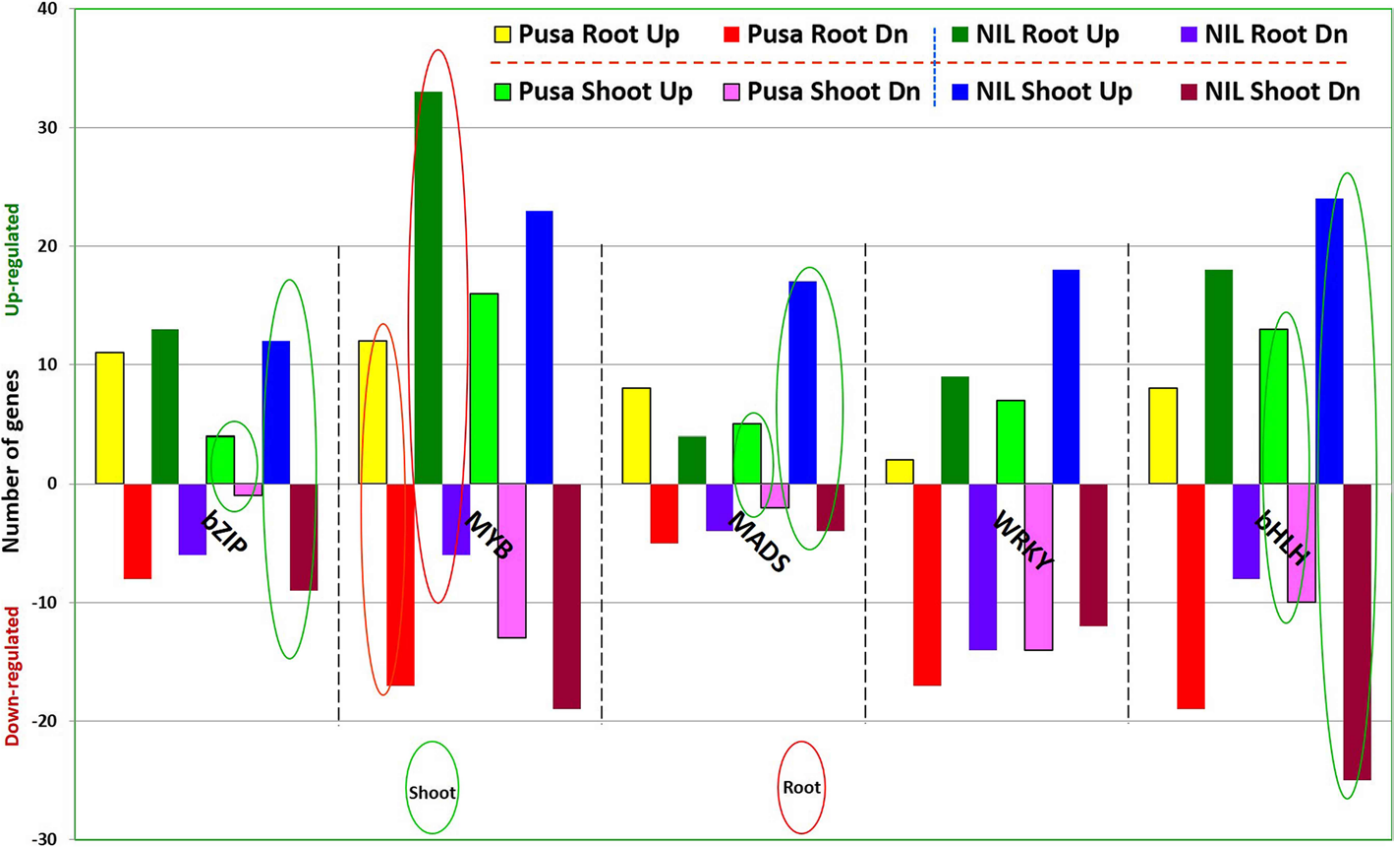
Differential expression of various transcription factor family genes in shoot and root of the stress-sensitive (Pusa-44) and stress-tolerant (NIL-23) rice genotypes grown hydroponically under P-starvation stress. Shoot and root tissues were collected from 45-day-old plants, continuously grown under control (16 ppm inorganic phosphorus) or treatment (0 ppm Pi), and used for whole transcriptome analysis

Expression analysis of transcription factors (TFs) in root and shoot of the rice genotypes under P-starvation stress revealed that in root of NIL-23 most of the TFs (210 out of 275) were up-regulated n root. Similarly, in shoot of NIL-23 more number of TFs (277 out of 389) were observed to be up-regulated. Among the differentially expressed TFs, some of the well-known TFs showing significantly up-regulated expression in NIL-23 under the stress include *bZIP*, *MYB*, *WRKY*, and *bHLH* (Fig. 7). More interestingly, a homeobox domain containing protein (LOC_Os03g51690) was observed to be considerably up-regulated (8.03-fold) in root and down-regulated (− 5.09-fold) in shoot of NIL-23, but its expression was not detected in Pusa-44. Similarly, another homeobox domain containing protein (LOC_Os06g36680), auxin response factor (LOC_Os07g08520), and AP2 domain containing protein (LOC_Os03g15660) were up-regulated in root and shoot of NIL-23. Moreover, the homeobox protein knotted-1 (LOC_Os03g51710) was exclusively expressed (6.28-fold up-regulated) in root of NIL-23. Similarly, GRAS family transcription factor containing protein (LOC_Os11g04570), histone-like transcription factor (LOC_Os01g39850), AP2 domain containing protein (LOC_Os04g46410), and HSF-type DNA-binding domain containing protein (LOC_Os06g36930) were exclusively expressed (> 2.5-fold up-regulated) in root of NIL-23 (Supplementary Table S5).

### GO analysis of differentially expressed genes

To gain insights into the DEGs in the contrasting rice genotypes under the stress, gene ontology (GO) analysis was performed, and their role in diverse biological/cellular/molecular processes was deciphered. GO analysis for root of NIL-23 indicated a larger number of genes to be up-regulated which were associated with a considerably higher number (229) of GO terms, while smaller number of genes were down-regulated and associated with a lesser number (134) of GO terms. GO analysis for shoot of NIL-23 indicated that up-regulated genes were associated with 131 GO terms, while down-regulated genes were associated 199 GO terms. On the other hand, the up- and down-regulated genes in shoot of Pusa-44 were associated 212 and 84 GO terms, respectively.

**Fig. 8:**
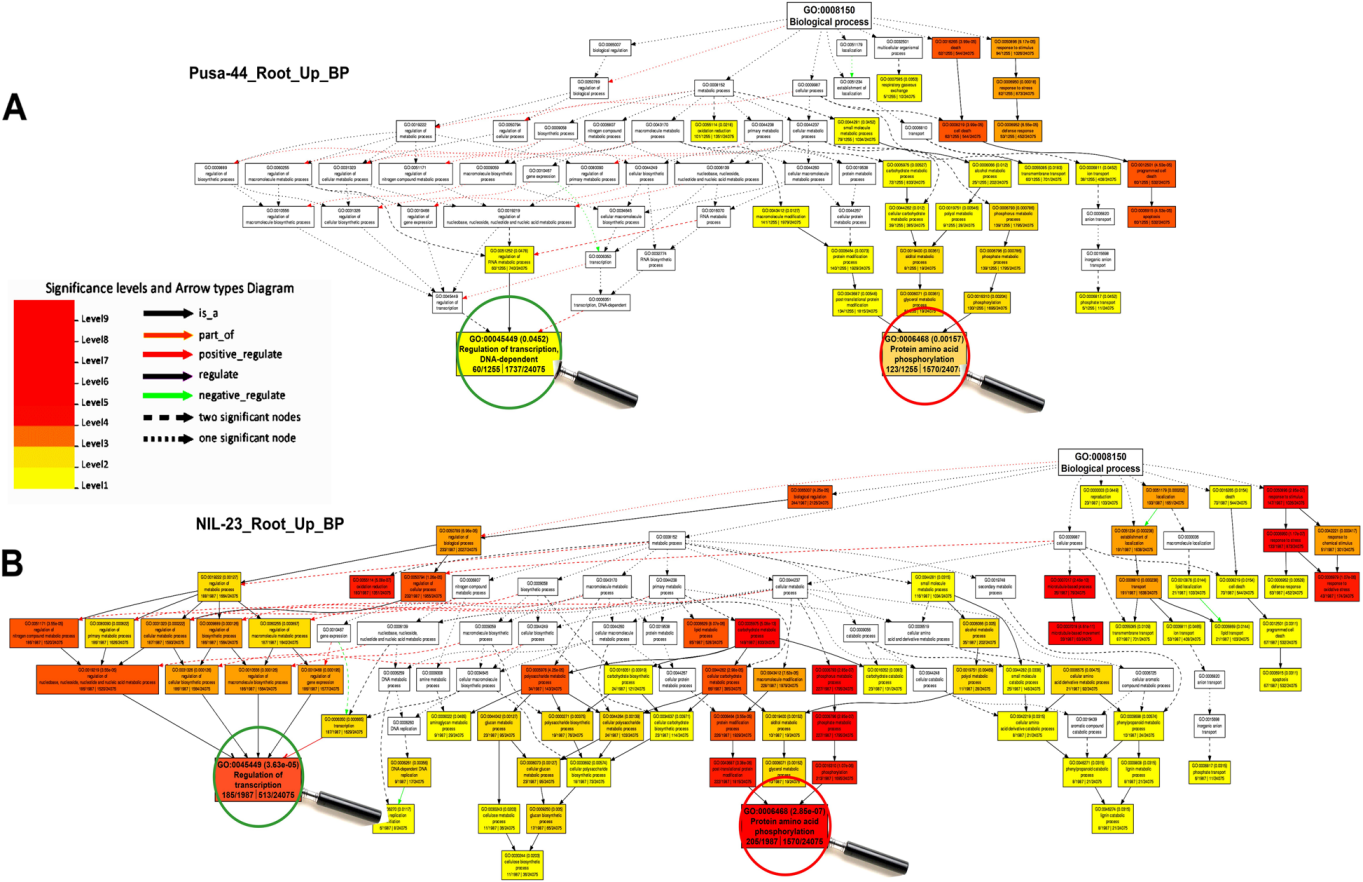
Gene ontology (GO) analysis of biological processes under P-starvation stress in roots of contrasting rice genotypes. **A** Over-represented GO terms in stress-sensitive Pusa-44, and **B** over-represented GO terms in stress-tolerant NIL-23 rice genotype

**Fig. 9:**
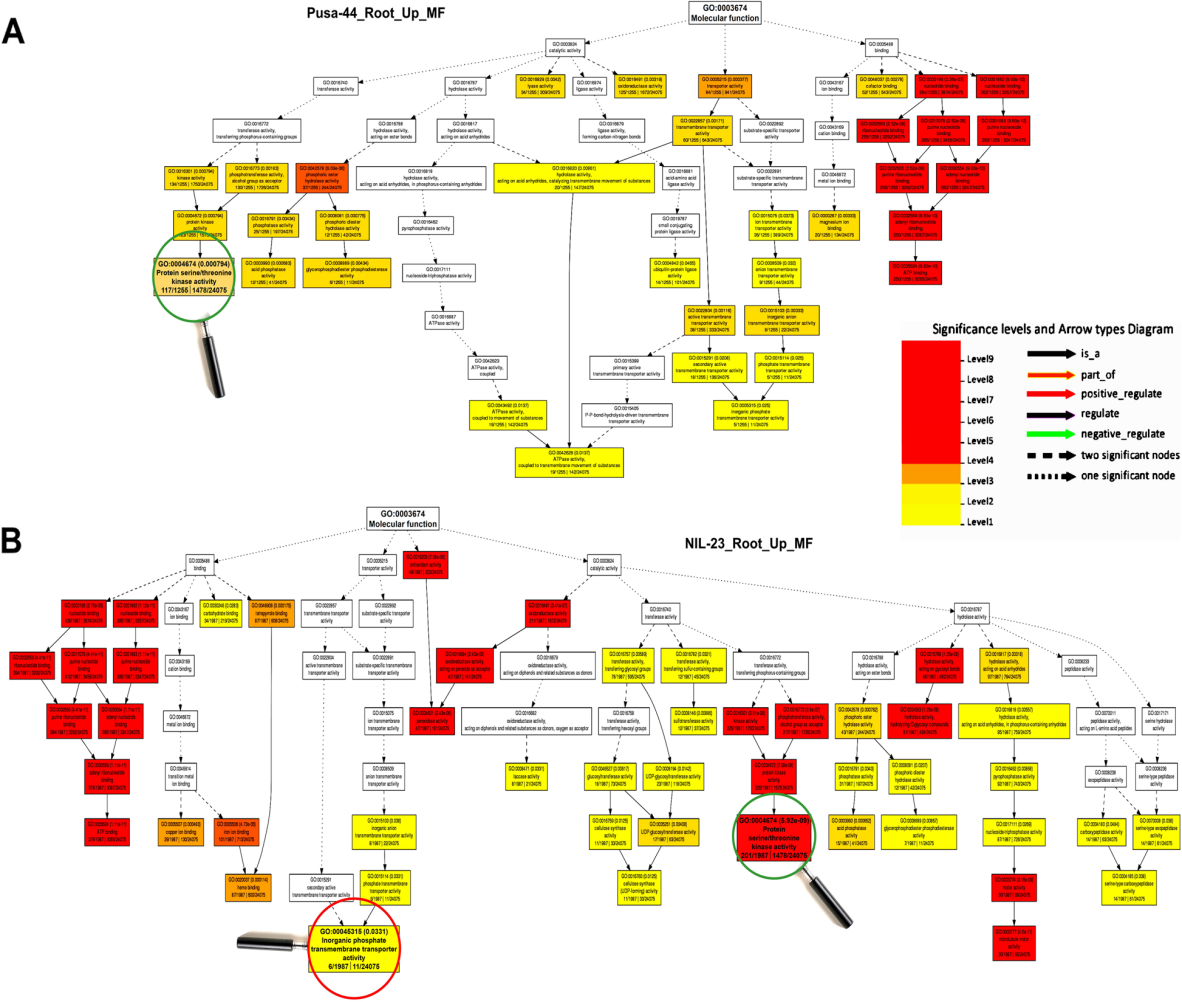
Gene ontology (GO) analysis of molecular functions under P-starvation stress in roots of contrasting rice genotypes. **A** Over-represented GO terms in stress-sensitive Pusa-44, and **B** over-represented GO terms in the stress-tolerant NIL-23 rice genotype

Under P-starvation stress, the significantly enriched GO terms belong to molecular functions, suggesting that molecular functions play most important roles in rendering P-starvation stress tolerance. Moreover, biological processes including regulation of gene expression, response to abiotic stress, antioxidant activity, transporter activity, nucleic acid binding TF activity, catalytic activity, and chromatin structure were significantly affected under the stress (Fig. 8). In depth GO analysis of the DEGs revealed dynamic modulation of molecular function, such as phosphate homeostasis, DNA replication, transcriptional/post-transcriptional gene regulation, redox homeostasis, to improve the stress tolerance (Fig. 9). In addition, the genes involved in carbohydrate and lipid metabolism, nitrogen metabolic processes, photosynthesis, were observed to be modulated by the stress. Epigenetic regulation of gene expression and post-translational protein modification were the other important GO terms over-represented.

### Differential expression of cell wall associated genes

In depth analysis of the DEGs suggested that a subset of genes are involved in cell wall and associated activities during P-starvation stress. Such genes include glycine-rich cell wall structural protein and expansins that showed considerably up-regulated expression in root of NIL-23 during the stress (Supplemental Table S6). The stress caused up-regulated (~ sevenfold) expressions of glycine-rich cell wall structural protein gene (LOC_Os10g31540) in roots of NIL-23, while it was down-regulated (~ threefold) in roots of Pusa-44. Expression of expansin precursor gene (LOC_Os10g40730) was up-regulated (~ 3.8-fold) in roots of NIL-23, while down-regulated (~ 2.5-fold) in roots of Pusa-44. Similarly, expressions of CESA7-cellulose synthase (LOC_Os10g32980) was up-regulated (~ 4.6-fold) in roots of NIL, while down-regulated (~ 1.4-fold) in roots of Pusa-44. In addition, several other genes, including glucan endo-1,3-beta-glucosidase precursor (LOC_Os07g35520), microtubule associated protein (LOC_Os01g49200), extra-large G-protein-related (LOC_Os02g15820) and tetraspanin family protein (LOC_Os06g44310), were observed to be up-regulated in roots of NIL-23, but down-regulated in roots of Pusa-44.

Some of the LTPL-Protease inhibitor family proteins (e.g. LTPL143, LOC_Os10g40480; LTPL139, LOC_Os10g40430) were observed to be considerably (4 to eightfold) up-regulated in roots of NIL-23, whereas these were down-regulated (2 to eightfold) in roots of Pusa-44 under P-starvation stress. Moreover, expressions of aquaporin (LOC_Os03g05290) and ABC transporter, ATP-binding protein (LOC_Os05g04610) were also observed to be up-regulated in roots of NIL-23, whereas down-regulated in roots of Pusa-44 under the stress (Supplementary Table S6).

### Differential expression of genes for phytohormone and signal transduction

Expression of auxin-responsive protein (LOC_Os05g48270) was observed to be up-regulated (∼5.82-fold) in roots of NIL-23, while down-regulated (∼2.13-fold) in Pusa-44 under P-starvation stress. Similarly, an auxin-induced protein 5NG4 (LOC_Os01g36580) was observed to be highly (5.69-fold) up-regulated in roots of NIL-23, but down-regulated in roots of Pusa-44. Other auxin-responsive family genes (like LOC_Os12g40900, LOC_Os12g40890, LOC_Os02g05050, LOC_Os02g13520) were also observed to be up-regulated in roots of NIL-23 but down-regulated in Pusa-44 under the stress (Supplementary Table S7). Moreover, the genes for auxin efflux carrier component (e.g. LOC_Os06g44970, LOC_Os02g50960) were also observed to be up-regulated in roots of NIL-23, whereas down-regulated in roots of Pusa-44 under P-starvation stress.

Expression of the genes involved in synthesis or response to other phytohormones, like jasmonate-induced protein (LOC_Os04g22900) and gibberellin 2-β-dioxygenase (LOC_Os05g48700) were also observed to be up-regulated (∼5.98 and ∼4.52-fold, respectively) in roots of NIL-23, but down-regulated in roots of Pusa-44 under P-starvation stress (Supplementary Table S7).

### Differential expression of genes involved in carbohydrate and lipid metabolism

Some of the genes involved in carbohydrate and lipid metabolism were observed to be differentially expressed in root of the contrasting rice genotypes. The gene for glycerophosphoryl diester phosphodiesterase (LOC_Os03g40670) associated with cell wall organization was observed to be up-regulated in roots under P-starvation stress, particularly in NIL-23. Other genes like glycosyl hydrolase (LOC_Os05g15770), lactate/malate dehydrogenase (LOC_Os08g33720), enolase (LOC_Os03g14450), phosphoenolpyruvate carboxylase (LOC_Os08g27840), pyruvate kinase (LOC_Os11g05110), glyceraldehyde-3-phosphate dehydrogenase (LOC_Os08g03290) were also observed to be up-regulated in roots of NIL-23 under the stress. However, genes for trehalose-6-phosphate synthase (LOC_Os09g20990), sucrose synthase (LOC_Os06g09450), and asparate aminotransferase (LOC_Os09g28050) were observed to be down-regulated in roots of the contrasting rice genotypes under the stress (Supplementary Table S8).

### Differential expression of photosynthesis related genes

Expression level of the gene for photosystem II (44 KDa) reaction center (LOC_Os04g16874) was observed to be down-regulated in root of both the rice genotypes, particularly in NIL-23 (− 5.95-fold) under P-starvation stress. Similarly, oxygen-evolving enhancer protein 1 (LOC_Os01g31690), cytochrome b6 (LOC_Os10g21324), and ATP synthase subunit alpha (LOC_Os04g16740) were also observed to be down-regulated in roots of the rice genotypes under the stress. Genes for the other components of photosynthesis machinery, like NADPH-dependent oxidoreductase (LOC_Os10g21418), chlorophyll A-B binding protein (LOC_Os06g21590), and photosystem II P680 chlorophyll A apoprotein (LOC_Os10g21310), were also observed to be significantly down-regulated in root tissues of both the rice genotypes. (Supplementary Table S9). On the other hand, the genes for photosystem II P680 chlorophyll A apoprotein (LOC_Os10g21310) and cytochrome b6 (LOC_Os10g21324) were observed to be up-regulated in shoot of NIL-23 under the stress.

### Differential expression of genes involved in epigenetic regulation

Many of the genes involved in epigenetic regulation of gene expression were observed to be differentially expressed. Some of the important genes involved in histone-modification include core histone H2A/H2B/H3/H4 domain containing protein (LOC_Os10g28230, LOC_Os03g06670, LOC_Os03g02780) were up-regulated in root of NIL-23 (stress tolerant genotype), down-regulated in Pusa-44 (stress sensitive genotype) under P-starvation stress (Supplementary Table S10). Moreover, the gene for jmjC domain containing protein (LOC_Os02g58210), involved in histone-demethylation, was observed to be 1.44-fold up-regulated in roots of NIL-23 under the stress, while it was down-regulated (− 1.74-fold) in Pusa-44 rice genotype. Expression of C-5 cytosine-specific DNA methylase (LOC_Os10g01570) was observed to be up-regulated (∼3.33-fold) in roots of the NI-23 under the stress, whereas it was down-regulated (2.48-fold) in roots of Pusa-44. Moreover, the gene for methyl-CpG binding domain containing protein (LOC_Os12g42550) was observed to be down-regulated in roots of the stress tolerant rice genotype. Interestingly, the genes for hsp20-α-crystallin family protein (LOC_Os03g06170, LOC_Os10g07210, LOC_Os10g07200), low-molecular-weight (20 kDa) protein produced under heat-shock or other environmental stress, were observed to be up-regulated (2.8 to 5.5-fold) in roots of NIL-23 under the stress, while they were down-regulated (2.9 to 3.9-fold) in roots of Pusa-44 (Supplementary Table S10).

### Validation of transcriptome data by Real-Time PCR analysis

**Fig. 10:**
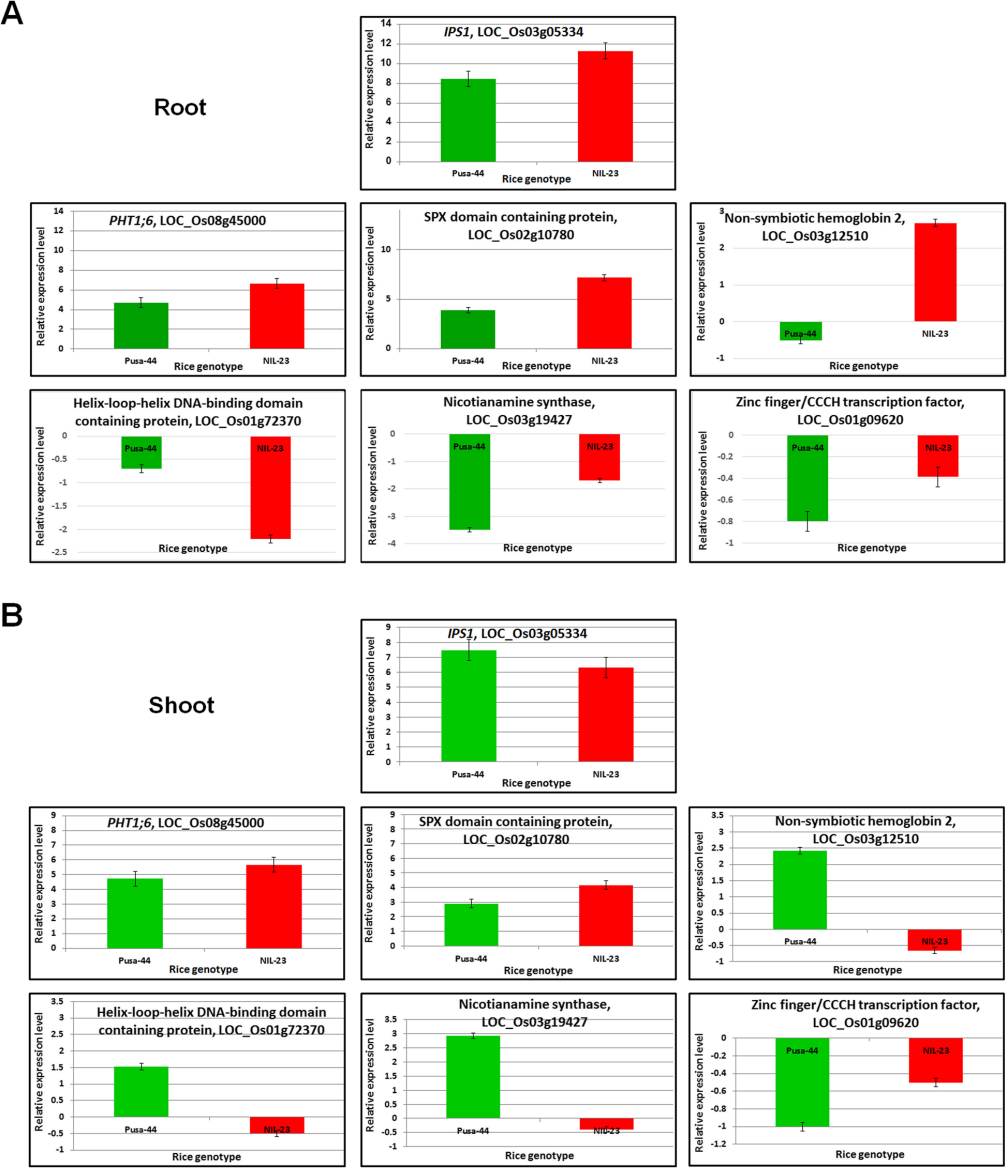
RT-qPCR validation of seven randomly selected differentially expressed genes. cDNA was prepared for the tissues collected from 45-day-old plants of P-deficiency stress-sensitive (Pusa-44) and stress-tolerant (NIL-23) rice genotypes grown hydroponically under control (16 ppm inorganic phosphorus) or treatment (0 ppm Pi). Data represent the mean ± SD (n = 3)

To validate the DEGs in the contrasting rice genotypes, expression level of seven randomly selected genes was validated in root and shoot in response to P-starvation stress by RT-qPCR analysis. The results were consistent with the expression pattern of the genes detected by RNA-seq. (Fig. 10). Thus, the RT-qPCR results confirmed trustworthy of the data we obtained from RNA-seq.

## Discussion

Inorganic phosphorus is often a limiting factor for plant growth, development, and productivity. However, plants take adaptive measures like altering root growth/architecture to access a larger volume of soil in search of P, excretion of organic acids/phosphatases/nucleases to solubilize Pi from organic sources and/or recycle internal Pi [2] to mitigate the effects of stress. Though some of the components of P-deficiency signaling in plants have been identified, the major pathways are still unexplored. *PSTOL1*, coding for a *Pup1*-specific protein kinase [29], was identified to impart variation in P-deficiency tolerance among the modern sensitive rice varieties and the tolerant (Kasalath) genotype. Most of the investigations on P-deficiency tolerance in plants were carried out at the seedling stage [20–25, 58] as plant-stand establishment is the first and one of the important steps for crop productivity. Once the seedlings are established, requirement of P for the growth and development (vegetative growth) of the plants is equally important. During the vegetative stage, plants grow rapidly and the requirement of P is considerably higher than that required during seedling stage. Hence, sevearl biochemical, physiological and molecular strategies are adopted by the plants to meet the requirements [59].

Therefore, we aimed at investigating the strategies/mechanisms adopted by rice plants to cope up with the limited availability of Pi beyond the seedling stage of growth. When a plant grows in P-deficient soil, it faces a continueous stress starting from germination through tillering until maturity. Hence, the rice plants were grown under P-starvation stress starting from the germination. Moreover, zero Pi in the hydroponic medium provides the most contrasting condition with respect to the concentration of Pi (16 ppm) supplied, which has been reported to be the best Pi concentration in PusaRicH medium for growth of a large number of rice genotypes in hydroponics [60]. Therefore, we used no/zero (0 ppm) Pi in the hydroponic medium for the stress treatment.

### Morphophysiological and biochemical adaptations in plant

The comparative morphological analyses of rice plants grown in P-sufficient and P-starvation conditions indicated a significant reduction in the number of roots but considerable increase in the length at seedling stage (20-day-old) of plant due to P-starvation. However, the tolerant genotypes showed more (50 − 60%) increase in the root length (Supplementary Fig. S1), which is in agreement with the earlier findings in different plant species [11, 25, 28, 35, 61]. This enables better Pi acquisition by increasing the root surface area for P absorption under the stress. Based on a pilot experiment, indicating no significant improvement in plant morphology with a higher than 16 ppm Pi concentration in the medium (Supplementary Fig. S2), the upper limit of Pi in the hydroponic medium was determined. Hence, 16 ppm Pi in PusaRicH medium was used to grow the rice plants under control condition. Further, a significant effect of P-starvation was observed on root and shoot development in all the three rice genotypes (Supplementary Fig. S3).

As P is one of the essential nutrients for living organisms, required for nucleic acids and phospholipids biosynthesis, enzyme activity, signal transduction, energy transport in the form of ATP, and several other metabolic processes, P-starvation considerably affects root and shoot growth in terms of reduced biomass production. At tillering stage (45-day-old plants), 71% reduction in height of Pusa-44 plants was observed when grown under P-starvation (0 ppm Pi) stress compared to that in the plants grown under control condition. Such reduction in height of NIL-23 and Kasalath plants was recorded to be only 47 and 51%, respectively (Fig. 1, Supplementary Fig. S4). Leaf being one of the most important parts of the plant for growth and development, P-starvation caused considerable reduction in size (51% reduction in stress-sensitive genotype, and 41 − 44% reduction in the stress-tolerant genotypes) as well as surface area (84% reduction in stress-sensitive genotype, and 68 − 70% reduction in the stress-tolerant genotypes) of the leaf (Supplementary Fig. S5). As expected, we observed a significant reduction in chlorophyll content with decreasing availability of Pi in the hydroponic medium. However, the reduction was considerably higher when Pi was not present in the medium (Supplementary Fig. S6). P-deficiency was reported to affect chlorophyll content in leaf [62]. Considering the importance, chlorophyll *a* fluorescence analysis was proposed to be used to detect P-deficiency in soil under field condition [63].

For a more comprehensive understanding, the effect of P-starvation on growth of root, shoot and root − shoot biomass ratio was analyzed. This revealed a significant effect of P-starvation on growth of root (number/biomass, spread, and branching/tertiary root/root-hairs), particularly in Pusa-44 (Fig. 2, Supplementary Table S1). Plants deploy several strategies to acquire P, with emphasis on exploration and uptake of Pi from soil [64]. This is generally achieved by increasing root growth, which results in increased root − shoot biomass ratio. However, the increased root growth requires a large proportion of daily photosynthate, which may be further constrained due to the limited supply of Pi [64]. Our findings are in agreement with those of Péret et al. [65]. The increased root − shoot biomass ratio has been reported in tomato, onion maize, and sorghum [24, 25, 65, 66]. Moreover, our observations on increased root length and tertiary roots/root-hairs corroborate with the earlier findings [25, 28]. However, a significant reduction in spread of roots was observed due to P-starvation stress (Fig. 2B). The length of tertiary roots/root-hairs increased significantly due to the stress (Fig. 2C), which helps improving P acquisition by the plants. Nguyen and Stangoulis [61] proposed a change in RSA to be an indicator of P-deficiency tolerance in wheat. In certain plant species, root-cluster (proteoid roots) formation has also been reported in response to P-deficiency. Such specialized roots secrete organic acids, which acidify the soil and chelate metal ions around the roots [13]. This might be one of the reasons for the observed reduction in pH of the hydroponic medium (Supplementary Table S2).

A considerable increase in the production of acid phosphatases in roots (Supplementary Figure S7) and their secretion in the soil were observed, which help mobilizing the P present in fixed forms. Phosphatases, ribonucleases and organic acids release Pi from organic/inorganic compounds in the rhizosphere. The up-regulated expression of phosphocholine phosphatase (Os01g52230) and ser/thr phosphatase (Os10g02750) genes under P-starvation corroborates with findings of Mehra et al. [11]. Increase in APase activity was observed in roots, which increased with the decreasing Pi content in the hydroponic medium. Increase in APase activity was reported earlier by Yugandhar et al. [67] in rice under P-deficiency stress. Up-regulated expression of the genes coding for enzymes/proteins responsible for organic acid synthesis and exudation under P-deficiency stress has been reported earlier [68–70]. Moreover, the observed up-regulated expression of the genes involved in glycolysis is necessary to increase carbon supply for organic acid synthesis. Our observation on > 6.3-fold and > 2.4-fold up-regulated expression of LOC_Os08g33710 and LOC_Os01g67190 (the ribonuclease T2 family domain containing proteins), respectively, is in agreement with the findings of Gho et al. [17]. Increased excretion of organic acids and Pi-releasing enzymes such as RNases and purple acid phosphatases (PAPs) results in larger rhizospheric Pi pool for uptake. The increased roots/root-hairs enhance porosity and oxygen release by the plant resulting in oxidation of iron and release of protons, which cause increased rhizospheric acidity to solubilize soil P. Secretion of purple acid phosphatases (PAPs), like AtPAP10, AtPAP12, PvPAP1 and PvPAP3 has also been reported earlier to help releasing Pi and organic P like ADP, glycerol-3-P, dNTPs in Arabidopsis and *Phaseolus vulgaris* [71]. Besides, overexpression of *WRKY1* from *Gossypium barbadense* in Arabidopsis was reported to increase accumulation of acid phosphatases, the number of lateral roots, and P-content in root [72].

P-content in plant tissues was observed to increase with increasing availability of Pi in the hydroponic medium (Supplementary Fig. S9). More P in shoot compared to that in root, and higher P content with increasing Pi in the medium are in agreement with the findings reported by Yugandhar et al. [65] in rice. We observed better P acquisition by roots of the stress-tolerant genotypes even at lower P in the hydroponic medium. This resulted in higher P content in root and shoot of NIL-23 and Kasalath (stress-tolerant genotypes) compared to that in Pusa-44 (stress-sensitive genotype). Thus, these morphophysiological and biochemical traits are influenced by environment and controlled by various molecular regulators, which interact synergistically to increase P acquisition [73]. For example, our molecular data clearly indicate that the key genes involved in glycolytic bypasses were significantly up-regulated in NIL-23; thus helped manage the demand of Pi very efficiently even under P-starvation stress in association with other synergistic strategies.

### Molecular adaptations under P-starvation

In addition to the morphological, physiological and biochemical adaptations, plants implement molecular approaches to cope up with the P-deficiency stress by modulating gene expression. DEGs analysis in root and shoot tissues of the contrasting rice genotypes in response to P-starvation stress revealed that up-regulation of gene expression play major role in stress tolerance. The number of up-regulated genes was comparatively higher in both shoot and root of the P-deficiency tolerant (NIL-23) genotype (Fig. 3). With a similar number of total DEGs, significantly higher number of up-regulated genes and lower number of down-regulated genes in root of NIL-23, compared to that in Pusa-44, might be responsible for the observed P-starvation tolerance in NIL-23. Moreover, considerable increase in the number of up-regulated as well as down-regulated genes in shoot of NIL-23 helps managing growth of plant under P-starvation stress. The uniquely up-regulated (2402) genes in root of NIL-23 were ~ 1.6 times more than that in Pusa-44. Similarly, the uniquely up-regulated (4964) genes in shoot of NIL-23 were ~ 3.7 times more than that in Pusa-44. More importantly, the uniquely down-regulated genes (2626) in shoot of NIL-23 were ~ 3.6 times higher than that in the shoot of Pusa-44 (Fig. 4). The exclusively up-regulated (1695) genes in root of NIL-23 were ~ 1.9 times higher than that in root of Pusa-44. Similarly, the exclusively up-regulated (3892) genes in shoot of NIL-23 were ~ 4.3 times higher than that in shoot of Pusa-44 (Fig. 5A). The exclusively down-regulated (1743) genes in shoot of NIL-23 were observed to be ~ 3 times higher than that in shoot of Pusa-44 (Fig. 5B). interestingly, out of the 76 phosphorus-responsive marker genes identified from earlier studies in Arabidopsis and rice [33], 69 genes were up-regulated in the root of NIL-23 (67 in case of stress-tolerant genotype) under the stress, which validates the experimental set up as well as quality/fidelity of data/analysis (Fig. 6, Supplementary Table S3).

Transcriptome data indicated up-regulated expression of some of the RSA genes like glycosyl transferases, expansins, and xyloglucan galactosyl transferases in roots of NIL-23 (Supplementary Table S11). Increased activity of high-affinity P transporters during P-starvation has been reported to play important role in acquisition of Pi in plants [74]. Under P-starvation, expression of phosphate transporter 1 (*PHT1*) group genes for high-affinity P transporters is induced to increase the ability of roots in acquiring Pi from soils and mobilizing Pi within plants [75]. In the present study, differential expression of 13 *OsPHT1s* in root/shoot of the contrasting rice genotypes was observed under P-starvation stress (Supplementary Table S4). Expression of *OsPHT1;2* (a low-affinity Pi-transporter) was reported to increase under P-deficiency stress [41], suggesting its role in transport of Pi from roots to shoots. Similarly, *OsPHT1;6* (a high-affinity Pi-transporter) was reported to function in acquiring Pi from the soil [41]. We observed increased expression of *OsPHT1;6* in roots under P-starvation, highest (12.65-fold) in NIL-23; thus, our observation corroborate with the finding of Ai et al. [41] We observed root-specific expression of *OsPHT1;3*, *OsPHT1;5* and *OsPHT1;9*, which suggest their function in acquisition of Pi from soil. *OsPHT1;4*, coding for a plasmalemma-localized Pi-transporter, was reported to express in root and leaf of rice for acquisition and transport (homeostasis) of Pi [76, 77]. Our findings corroborate with the earlier reports. The up-regulated expression (9.02-fold) of *OsPHT1;13* observed in shoot of NIL-23 might be mainly responsible for mobilization of Pi from root to shoot, while it was observed to have major function (10.62-fold up-regulated expression in root under the stress) in acquisition of P from soil in Pusa-44. Moreover, *OsPHT1;12* expression was up-regulated in shoot of both the genotypes compared to that in roots (Supplementary Table S4), which might help mobilizing Pi from root to shoot. Remobilization of Pi within the plant was reported during vegetative and reproductive stage of plant growth [63]. A number of P transporter genes like *PHT1;3*, *PHT1;8*, *PHT1;10*, and *PHT1;13* were reported to be induced by nitrate availability [78].

TFs are important regulators of gene expression process, and they have been reported to play key roles in tolerance to various abiotic stresses. Homeobox-domain containing proteins have been reported to bind DNA and regulate transcription of the genes [79]. In the present study, expression of homeobox-domain containing protein and homeobox protein knotted-1 was up-regulated in root of NIL-23 that corroborates with the earlier findings [79]. Likewise, findings of the present study also showed that auxin response factor, AP2 domain containing proteins, GRAS family transcription factor containing protein, histone-like transcription factor, and HSF-type DNA-binding domain containing protein exclusively expressed (> 2.5-fold up-regulated) in root of NIL-23 (Supplementary Table S5) which is in agrement with the earlier findings [33, 34]. A P-deficiency-responsive MYB TF was reported earlier to over-express in Arabidopsis under P-starvation [80], which substantiate our finding. Similarly, other P-deficiency-responsive TF families like NAC, AP2, zinc finger, and WRKY [34, 81, 82] were also observed to be differentially expressed in the contrasting rice genotypes under P-starvation (Supplementary Table S5). Though bZIP, MADS, and bHLH TF family genes played important roles in shoot of NIL-23, differential expression of MYB family genes played more important roles in root of NIL-23 (Fig. 7). On the other hand, WRKY family genes were observed to play equally important roles in shoot and root for P-starvation tolerance. Various TFs including MYB, ERF/AP2, WRKY, CCAAT‐binding, Zinc finger, bHLH, and NAC have been reported to be over-expressed under P-deficiency stress [32, 83]. A bHLH TF was reported to play role in providing tolerance against P-starvation by improving RSA in rice [82].

Further insights in to the mechanisms involved in P-deficiency stress tolerance came from GO analysis, which deciphered the role of diverse biological/cellular/molecular processes under P-starvation stress tolerance. GO analysis in roots of NIL-23 revealed that a large number of up-regulated genes belong to 229 (more) GO terms, compared to only 175 (less) terms in roots of Pusa-44 (Fig. 8). Among these GO terms, regulation of transcription, protein/amino acid phosphorylation (biological process), and serine/threonine kinase activity, inorganic phosphate transporter activity (molecular function) played important roles in stress tolerance in NIL-23 (Figs. 8, 9). Up-regulated expression of dirigent genes in root (*At1g64160*) and leaf (*At2g21100*) has been reported in Arabidopsis under P-deficiency [83], which is in agreement with our finding. The Ser/Thr protein phosphatase family proteins modify other proteins by (de)phosphorylation, and regulate cellular functions, signal transduction, and responses to biotic and abiotic stresses [84]. Some of the cellular components like integral membrane component were over-represented (Supplementary Fig. S12), while others like oxygen evolving complex were under-represented (Supplementary Fig. S13) in roots of NIL-23. GO analysis in shoot of NIL-23 indicated exclusive over-representation of some of the biological processes like transcription process, amino acid phosphorylation (Supplementary Fig. S14), and molecular functions like serine/threonine kinase activity (Supplementary Fig. S16), cellular components like phosphofructokinase complex were observed to be exclusively enriched in the shoot of Pusa-44 (Supplementary Fig. S18). Similarly, GO analysis in shoot of NIL-23 indicated that a larger number of down-regulated genes belong to 199 GO terms (Supplementary Figs. S15, S17, S19).

**Fig. 11:**
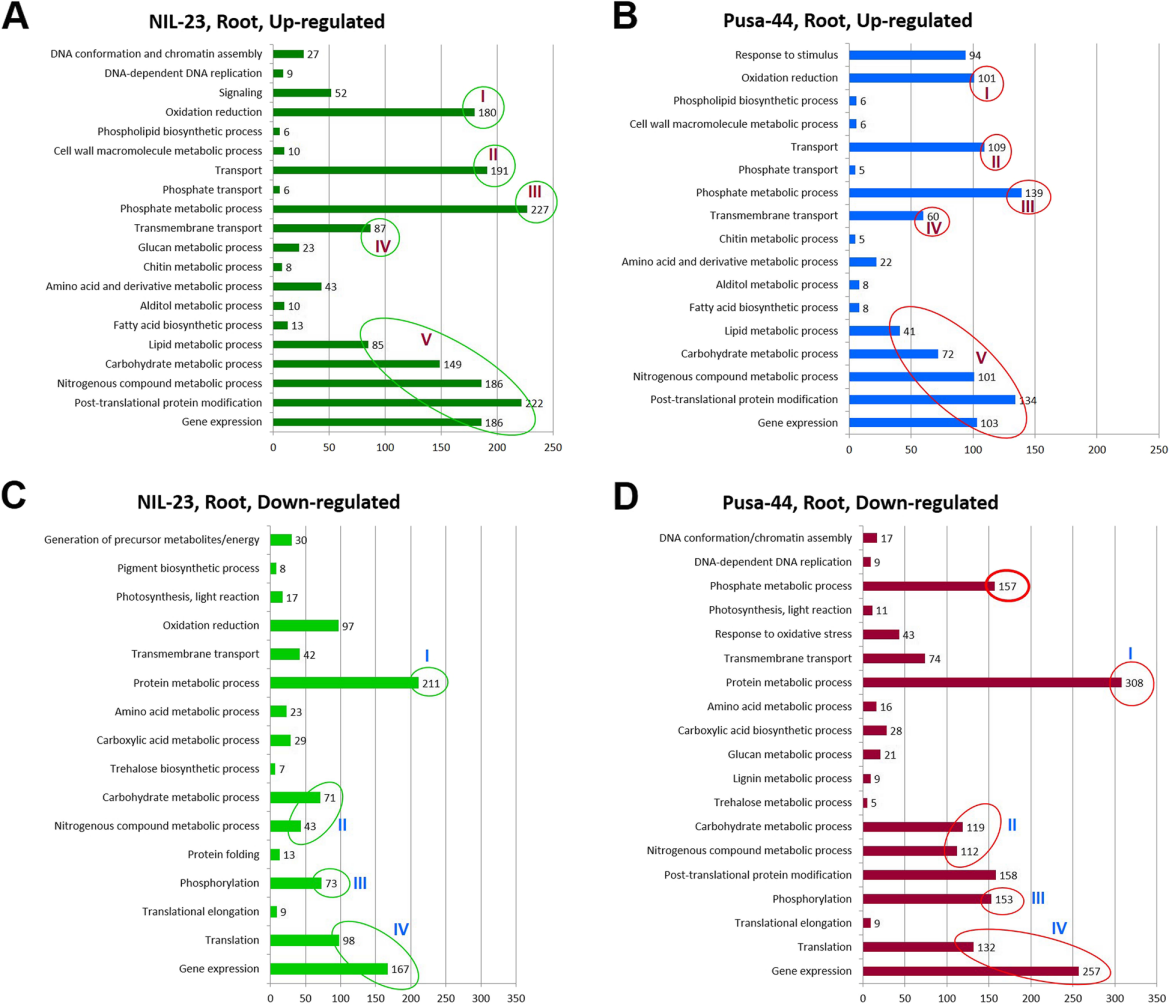
Differentially enriched GO terms and the number of respective genes up- or down-regulated in root of the contrasting rice genotypes grown under P-starvation stress. **A** The number of up-regulated genes for the GO terms in NIL-23, a stress-tolerant genotype, **B** number of up-regulated genes for the GO terms in Pusa-44, a stress-sensitive genotype, **C** number of down-regulated genes for the GO terms in NIL-23, **D** number of down-regulated genes for the GO terms in Pusa-44

The GO terms associated with carbohydrate, lipid, and nitrogen metabolism were enriched to improve nutritional status, energy supply, and cellular protection, particularly in NIL-23 (Fig. 11). Under P-starvation, cells bypass ATP or Pi-dependent enzymatic reactions of sugar metabolism by using Pi-independent pathways. The genes involved in such glycolytic bypasses were observed to be modulated under the P-starvation stress, particularly in root of NIL-23, which is in agreement with [11]. The first bypass, catalyzed by pyrophosphate-dependent phosphofructokinase (PPi-PFK), converts fructose-6 phosphate to fructose 1,6-bisphosphate without using ATP. PPi-PFK gene (*Os02g48360*) was up-regulated in NIL-23, while down-regulated in Pusa-44. The second bypass uses NADP-GAPDH to minimize Pi requirement needed by NAD-GAPDH to form 1,3-bisphosphoglycerate. The NADP-dependent aldehyde dehydrogenase encoding gene (*Os12g12590*) was up-regulated in root of NIL-23. Third bypass operates downstream in glycolysis by using phosphoenolpyruvate carboxylase (PEPC) and malate dehydrogenase (MDH) to replace pyruvate kinase, which require Pi. The PEPC (*Os09g14670*) and MDH encoding genes (*Os08g33720*) were induced by P-starvation (Supplementary Table S11). The GO terms for phosphorus metabolic process, transmembrane transport, and oxidation–reduction were significantly enriched in roots of NIL-23. Moreover, GO terms/genes for epigenetic regulation of gene expression and post-translational protein modification were differentially represented/expressed in root/shoot of NIL-23 (Supplementary Table S10).

Cell wall not only provides rigidity to plant, but also controls cell expansion. Therefore, cell often weakens the cell wall structure and maintains turgor/cellular integrity to achieve growth [85]. A subset of genes responsible for cell wall organization and associated activities exhibited up-regulated expression during P-starvation in NIL-23 (Supplementary Table S6). Among these, the genes encoding for expansins, LTPL-Protease inhibitor family proteins, and glycine-rich cell wall proteins, etc. were the important players that varied dynamically in roots under P-starvation stress. Expansin and LTPL-protease inhibitor family proteins are known to play important roles in cell wall loosening, wax/cutin deposition in the cell wall of expanding/multiplying cells. Plants deploy signaling mechanisms to sense the developmental and environmental cues and modulate the cell wall structure. Many auxin-responsive genes involved in cell wall biosynthesis and loosening were reported to be up-regulated in *siz1* mutant compared to that in the wild-type plants under P-starvation [86]. Our observations on up-regulated expression of auxin-responsive/induced genes support the roles of phytohormones in modulating the cell wall structure in roots of NIL-23 under P-starvation stress (Supplementary Table S7).

The genes involved in membrane-lipid remodeling have been reported to be induced by P-deficiency [87]. Lipid transfer proteins (LTPs), usually localized in extracellular spaces outside the plasma membrane, play important role in deposition/adhesion of wax and lipid barrier polymers. Signaling and tolerance to abiotic/biotic stresses are associated with LTPs [88]. In the present study, several lipid transfer protein (LTP) family genes were dynamically modulated for their expression during P-starvation stress in NIL-23. The genes for membrane lipid remodeling including like phospholipase C, phospholipase D, and glycerophosphodiester phosphodiesterases, were observed to be significantly up-regulated in NIL-23 compared to that in Pusa-44 under the stress. Pi pool of a cell can also be conserved by substituting phospholipids with sulfolipids and galactolipids in the membrane [89]. Phospholipids being one of the important components of biological membrane, P-deficient plant replaces some of the phospholipids with phosphorus-free galactolipid, sulfoquinovosyl diacylglycerol (SQDG), and digalactosyl diacylglycerol (DGDG) in order to minimize the dependence on Pi [11, 90, 91]. We observed up-regulated expression of monogalactosyldiacylglycerol (MGDG) synthase (*Os08g20420*) in root of both the rice genotypes, particularly NIL-23, which is in agreement of the findings of Mehra et al. [11] Thus, Pi deficiency is compromised by increased uptake of sulfur (S) which is clearly indicated by the increased activity of sulfur transporters. Genes involved in membrane-lipid alterations are known to get activated by P-deficiency [87]. Up-regulated expression of *SQD1* and *SQD2* genes, responsible for sulfolipid biosynthesis, was reported to be induced by P-starvation in Arabidopsis and rice [83, 92]. In the present study also, cell wall reorganization, phospholipid remodeling, and associated activity related genes exhibited up-regulated expression during P-starvation stress in NIL-23. Thus, P-starvation induced lipid remodeling, accumulation of MGDG and DGDG indicate that cellular lipid remobilization is one of the adaptive strategies for P-starvation tolerance in NIL-23, which is in agreement with the earlier reports [93, 94].

Modulation in metabolic processes, particularly sugar and lipid metabolism, has been reported to be crucial for adaptation to P-deficiency stress. Increase in sucrose biosynthesis in leaf of Arabidopsis, bean, barley, spinach, and soybean under the stress has been reported [95]. Alteration in diverse metabolic processes related to glucose, pyruvate, sucrose, starch, and chlorophyll under the stress has also been reported [11, 96]. Decrease in phosphorylated-sugars in leaf and root was reported under the stress because of reduced availability/activity of Pi/fructokinase/hexokinase [97, 98]. Down-regulated expression of trehalose − 6 − phophate synthase was reported under P-deficiency studies [81, 83], which corroborates with our findings. Pi has been also been reported to be involved in regulation of the distribution of fixed carbon between starch synthesis (in chloroplast) and transfer of triose-phosphate for sucrose synthesis (in cytoplasm) [99]. P‐deficiency was also reported to increase translocation of carbohydrate via phloem to roots to favor root growth for better acquisition of Pi from soil [100]. Thus, more efficient metabolic adjustments in NIL-23, compared to that in Pusa-44, are responsible better survival of NIL-23 under P-starvation stress. Phosphorus homeostasis between shoot and root under the stress has been reported to be maintained by modulating the expression of transporters, phosphatases, RNases, and the enzymes involved in metabolic processes [17, 69, 70, 83].

Photosynthetic process was reported to be inhibited under P-deficiency mainly because of the limiting effect of Pi for ATP synthesis, Rubisco activation, and RuBP regeneration in chloroplasts [6, 101, 102]. P-deficiency supresses Calvin cycle activity by reducing the amount and activity of Rubisco [103] and regeneration of ribulose-1,5-bisphosphate [102]. Moreover, phosphorus plays crucial structural and regulatory roles at the junction of photosynthesis, energy conservation, and carbon metabolism [104]. The balance between anabolic and catabolic carbon metabolism is disrupted under P-deficiency stress. The expression of the genes for photosystem (PS) I, PSII, Rubisco small subunits, Calvin cycle enzymes and chlorophyll A/B-binding proteins have been reported to be repressed by P-deficiency [105, 106]. At the same time, the genes involved in glycolysis, starch and sucrose synthesis (glucose − 6 − phosphate dehydrogenase, phosphofructokinase, frucose − 1,6 − bisphosphate aldolase, phosphoenolpyruvate carboxylase, glyceraldehyde − 3 − phosphate dehydrogenase, and sucrose transporters) have been reported to be up-regulated [68, 69], which corroborate with our findings. Such changes are necessary under Pi-deficiency to bypass dependency on the ATP- and Pi-dependent enzymes, and to modulate the metabolic processes required to generate energy and carbon skeleton [107, 108].

Photosynthesis being a most important photochemical sink for energy conversion, P-deficiency mediated inhibition of CO_2_ assimilation leads to damage to the photosynthetic apparatus by the excessive light/excitation energy. P-deficiency inhibits photophosphorylation process, and limits the availability of ATP/NADPH which affect metabolic processes. We observed ~ sixfold down-regulated expression of a gene (LOC_Os04g16874) for photosystem II reaction center under P-starvation in NIL-23 (Supplementary Table S9), which help protecting the plant from excessive excitation energy. The excessive excitation energy increases the production of reactive oxygen species (ROS); hence, non-photochemical quenching of ROS was reported to increase under P-deficiency in rice [21]. Our findings on differential expression of the genes involved in photosynthesis under stress are in line with that reported by Secco et al. [33]. Modulation in photosynthesis-related genes was observed even in roots of the rice genotypes under the stress, which is in agreement with Li et al. [22] and Wang et al. [36].

Phytohormones like auxin, abscisic acid, cytokinin, and gibberellins are known to play role in root development [109]. Auxin plays role in the development of lateral roots and thus helps acquisition of phosphorus. In Arabidopsis, cell cycle in lateral roots is regulated by auxin, and P-deficiency affects expression of the cell cycle genes like *CDKA, E2Fa, Dp-E2F* and *CyCD3*. Thus, modification in root architecture is associated with P-starvation through phytohormones. A linkage between auxin transporters PIN7 and APSR1 was reported because absence of ASPR1 decreased expression of *PIN7* under P-starvation [110]. In addition, P-starvation reduces gibberellic acid (GA) content leading to increased DELLA proteins [111], which resulted in increased length of root hairs in NIL-23 under the stress.

Chromatin architecture is a key determinant of gene expression in eukaryotes. Certain chromatin-related components have been reported to play roles in root hair growth during P-deficiency stress in Arabidopsis. A histone deacetylase (HDA19) was reported to get induced and increased root hair growth under P-deficiency in Arabidopsis [112]. Zahraeifard et al. [113] reported alteration in genome-wide H2A.Z distribution due to P-starvation in rice. Zhang et al. [114] suggested the role of nucleosome remodelers in modulating nucleosome occupancy and differential gene expression under P-deficiency stress. A possible modulation in chromatin architecture can be expected under the stress due to the observed differential expression of jmjC domain containing protein, core histone H2A/H2B/H3/H4 domain containing proteins, C-5 cytosine-specific DNA methylase, and methyl-CpG binding domain containing protein. An extensive remodeling of global DNA methylation under P-deficiency stress in Arabidopsis was reported by Yong-Villalobos et al. [115] Hypo- and hyper-methylation of DNA in the vicinity of TF binding sites under P-starvation in Arabidopsis was reported by Yong-Villalobos et al. [116] Our observation on DNA methylase to be up-regulated and methyl-CpG binding domain containing protein to be down-regulated in the stress tolerant genotype under the stress (Supplementary Table S10) corroborate with the above findings. Similarly, differential expression of P-starvation-responsive proteins like histone chaperone (in our case hsp20) and nucleosome assembly protein were reported in Arabidopsis under P-starvation stress [91].

Several QTLs have been detected on different chromosomes in rice for P uptake, among which *Pup1* is the major one. Although ectopic expression of *PSTOL1* in P-deficiency sensitive rice cultivar was found to enhance early root growth enabling plants to acquire more phosphorus, as well as other nutrients, and increase grain yield in P-deficient soil [29], the role of *Pup1* is not clear. We observed up-regulated expression of fatty acid oxygenases, dirigent proteins, aspartic proteinases, and protein kinases genes, particularly in root of NIL-23 under P-starvation stress (Supplementary Table S11). Since the genes on *Pup1* QTL do not code for a known P uptake-related protein, the mode of action of *Pup1* is still unclear. However, *OsPupK20*, *OsPupK29*, and *OsPupK46* (protein kinase genes) have been reported to be the candidate genes associated with root-specific functions [34, 117].

## Conclusions

**Fig. 12:**
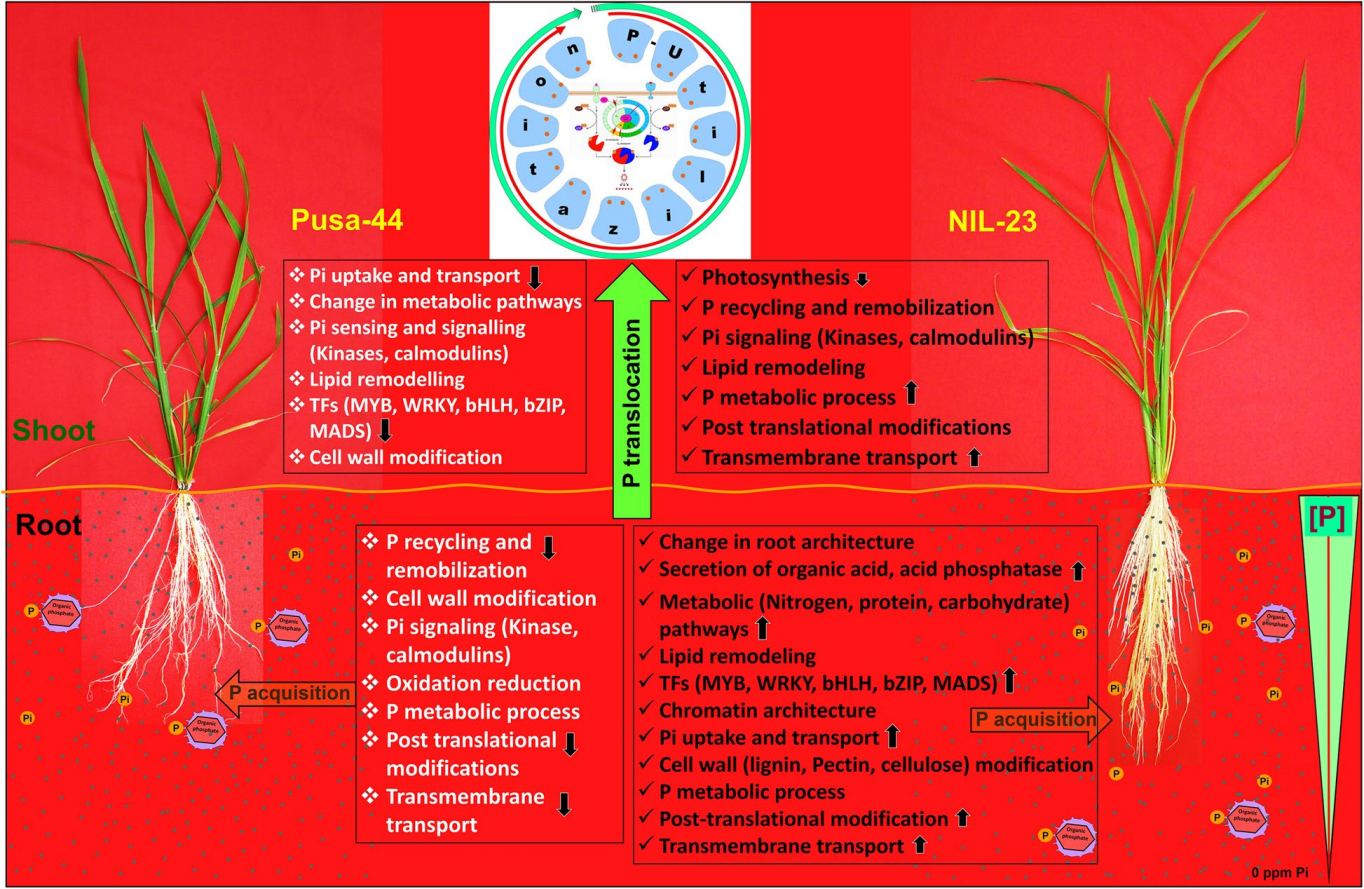
Diagrammatic representation of the mechanisms involved in P-deficiency stress tolerance in rice (Pusa-44, stress sensitive, and NIL-23, stress tolerant). The upward arrow (↑) indicates higher/supportive role of the mechanism, while the downward arrow (↓) indicates down-regulation of the process in under the stress

As P is the second most important macronutrient for the growth and development of plants, it is unfortunate that many aspects of P uptake, transport and use-efficiency in plants are not yet thoroughly understood. Some of the studies provide insights into P distribution, functional characteristics of the transporters, and differential expression of the genes involved in P-deficiency tolerance. The present study reveals significant roles of the transporters (OsPHT1;6, OSPHT1;10), signalling molecules like jasmonate/auxin-induced proteins (LOC_Os04g22900, LOC_Os01g36580), auxin-responsive protein (LOC_Os05g48270), LTPL-protease inhibitor family proteins (LOC_Os10g40430, LOC_Os10g40480), glycerophosphoryl diester phosphodiesterase family protein (LOC_Os03g40670), phosphatases (LOC_Os01g52230, LOC_Os08g17784, LOC_Os11g38050, LOC_Os07g01540, LOC_Os05g02310), transcription factors like homeobox domain containing protein (LOC_Os03g51690), MYB family TFs (LOC_Os03g62100, LOC_Os02g22020) and AP2 domain containing proteins (LOC_Os08g36920, LOC_Os10g11580), core histone domain containing protein (LOC_Os10g28230), and glycine-rich cell wall structural proteins (LOC_Os10g31530, LOC_Os10g31540) in providing P-starvation stress tolerance in rice. Better P-use efficiency and lower Pi demand of NIL-23, because of introgression of the *Pup1* QTL in the Pusa-44 genetic background, are responsible for better growth and survival of NIL-23 plants until vegetative stage even under P-starvation stress. The present study offers plentiful molecular information/candidate mechanisms for functional dissection of P-deficiency stress tolerance in rice, which might further help understanding the regulatory network by integrating the biochemical, physiological, genetic, and molecular mechanisms to enhance P-use efficiency in crop plants to cope with the limiting availability of P in the soil (Fig. 12), particularly because of the changing environmental conditions. Thus, the mechanisms like modulation in root system architecture, P acquisition, internal Pi remobilization, energy conservation might help improving P-use efficiency in rice through genetic and epigenetic approaches to improve productivity of modern cultivars in P-deficient soils.

## Methods

### Plant materials, growth conditions and P-starvation stress imposition

Two contrasting rice genotypes [Pusa-44 (P-deficiency sensitive) and NIL-23 (P-deficiency tolerant) received from the Division of Genetics, ICAR-Indian Agricultural Research Institute, New Delhi] were used in the present study. Mature seeds of the contrasting rice genotype [along with the original *Pup1* QTL donor (Kasalath), received from the Division of Genetics, ICAR-Indian Agricultural Research Institute, New Delhi] were used to raise seedlings/plants hydroponically in P-sufficient (16 ppm Pi), P-deficient (1 or 4 ppm) or P-starvation (0 ppm Pi) condition. More details of the rice genotypes (Supplementary Figs. S20, S21), growth conditions (Supplementary Fig. S22) and P-starvation stress have been provided in Supplementary Methods. Shoot and root tissues collected from 45-day-old plants (in vegetative/tillering stage) were used for various analyses.

### Estimation of APase activity, root − shoot biomass ratio, chlorophyll and phosphorus contents

Acid phosphatase (APase) activity in shoot and root tissues, secreted APase from roots, and Root − shoot biomass ratio were estimated/calculated. Total chlorophyll content in leaf was estimated using dimethyl sulfoxide method. Total phosphorus content in shoot and root tissues was determined using Vanadate-molybdate method. The detailed procedure of the above-mentioned analyses is provided as Supplementary Methods.

### RNA isolation, library preparation, and data analyses

To analyze the effects of P-starvation stress on gene expression in shoot and root of the contrasting rice genotypes, total of 16 libraries were prepared, and got sequenced commercially on Illumina platform using PE 2 × 150 bp chemistry. The raw sequence data was submitted to NCBI Sequence Read Archive (SRA) database under the BioProject ID PRJNA667189. Reference-based mapping of the RNA-seq data was performed using the rice reference genome (TIGR v7) with the help of Hisat2 and Stringtie package. The number of mapped clean reads for each gene was counted and normalized into the reads per kilo base per million (RPKM) value. Differentially expressed genes (DEGs) were annotated with gene ontology terms and key pathways via functional classification and Kyoto Encyclopedia of Gene and Genomes pathway mapping, respectively. DEGs were compared between control and treatment, as well as between the rice genotypes. Genes with log_2_ fold-changes > 1 (i.e., fold-change > 2) and *p* < 0.05 were considered differentially expressed. Detail procedures of RNA isolation, library preparation, and data analysis, etc. are provided in the Supplementary Methods.

### Validation of differentially expressed genes by RT-qPCR

To confirm the results of RNA-seq, seven DEGs (four up-regulated and three down-regulated) were selected randomly for quantitative (RT-qPCR) analysis following the MIQE guidelines. The data was analyzed through melt-curve to check specificity of the PCR amplification. The relative gene expression was determined by the 2^−ΔΔCt^ method. Actin and tubulin genes were used as reference/housekeeping genes. More details of the RT-qPCR validation are provided in the Supplementary Methods, and the primers used are listed in Supplementary Table S12.

This is confirmed that all the materials and methods used for the present research work complied with relevant institutional, national, and international guidelines and legislation.

## Supplementary Information

**Additional file 1: Supplementary Tables S1 to S2.** Comparative analysis of root-shoot biomass ratio in the contrasting rice genotypes along with the donor parent, Effect of the root-secreted acid phosphatase on pH of the hydroponic medium.

**Additional file 2: Supplementary Tables S3 to S11.** Expression level of the P-starvation-inducible genes, 13 Phosphorus transporters during P-starvation stress, Differentially expressed Transcription Factors, Cell wall associated genes in root of the contrasting rice genotypes, Phytohormone encoding genes, Genes associated with carbohydrate and lipid metabolism, Photosynthesis related genes, Genes associated with epigenetic regulation of gene expression, Master data-sheet of the genes detected for their expression in the contrasting rice genotypes under P-starvation stress.

**Additional file 3: Supplementary Table S12.** List of the primers used for RT-qPCR validation of the randomly selected differentially expressed genes (DEGs) in the contrasting rice (Pusa-44, P-deficiency stress sensitive; NIL-23, P-deficiency tolerant) genotypes.

**Additional file 4: Supplementary Methods.** Detailed method for the experiments performed.

**Additional files 5: Supplementary Figures S1 to S22.**

## Data Availability

The morphophysiological and biochemical data generated/analyzed during this study are included in the Supplementary files. RNA-sequencing (transcriptome) raw reads data are available at NCBI Sequence Read Archive (SRA) database (https://www.ncbi.nlm.nih.gov/sra) under the BioProject ID: PRJNA667189.

## Abbreviations

APase: Acid phosphatase
DEGs: Differentially expressed genes
FC: Fold change
FDR: False discovery rate
GA: Gibberellic acid
DGDG: Digalactosyl diacylglycerol
GO: Gene ontology
LTP: Lipid transfer protein
MGDG: Monogalactosyl diacylglycerol
MGDGS: Monogalactosyl diacylglycerol synthase
NIL: Near-isogenic line
PHT1: Phosphorus transporter 1
P: Phosphorus
Pi: Inorganic phosphorus
ppm: Part per million
PS: Photosystem
Pup1: Phosphorus uptake 1
QTL: Quantitative trait loci
RSA: Root system architecture
RuBP: Ribulose-1,5-bisphosphate
SQDG: Sulfoquinovosyl diacylglycerol
TF: Transcription factor
