# Supplementary material for: Characterization of contrasting rice (Oryza sativa L.) genotypes reveals the Pi-efficient schema for phosphate starvation tolerance
Source: BMC Plant Biol. 2021 Jun 21;21:282. doi: 10.1186/s12870-021-03015-4 (PMC8215752; doi:10.1186/s12870-021-03015-4)
Supplement: Supplementary file 3 — Additional file 3: Supplementary Table S12. List of the primers used for RT-qPCR validation of the randomly selected differentially expressed genes (DEGs) in the contrasting rice (Pusa-44, P-deficiency stress sensitive; NIL-23, P-deficiency tolerant) genotypes. [file 12870_2021_3015_MOESM3_ESM.doc]

**Characterization of contrasting rice (*Oryza sativa* L.) genotypes reveals the Pi-efficient schema for phosphate starvation tolerance**

**Suresh Kumar, Pallavi, Chetna Chugh, Karishma Seem, Santosh Kumar, K. K. Vinod, and Trilochan Mohapatra**

**Supplementary** **Table S12.** The list of primers used for RT-qPCR validation of the randomly selected differentially expressed genes (DEGs) in the contrasting rice (Pusa-44, P-deficiency stress sensitive; NIL-23, P-deficiency tolerant) genotypes.

| **Gene** | **Gene LOC** | **Forward Primer (5′→ 3′)** | **Reverse Primer (5′→ 3′)** |
| --- | --- | --- | --- |
| *IPS1* | LOC_Os03g05334 | TCCGGGAGAAGGTGTTCGAG | CCTCCTCTCCACCAACCATG |
| *PHT1;6* | LOC_Os08g45000 | GCCCCTGCAAACTGTACTG | AGCCAGGCCAGTTATATATCAAC |
| SPX domain containing protein gene | LOC_Os02g10780 | TCACCACCGACCTCCTCTAC | GGCTTTGAACCCTTCTCCTC |
| Helix-loop-helix DNA-binding domain containing protein gene | LOC_Os01g72370 | CCCAGTACACCAGCTTCACC | TCTTCTTCCATCTGCAAGCTC |
| Nicotianamine synthase gene | LOC_Os03g19427 | TGATCAACTCCGTCATCGTC | GCCATAATATAGTGCGCCTTTC |
| Zinc finger/CCCH transcription factor gene | LOC_Os01g09620 | TCCGGGAGAAGGTGTTCGAG | CCTCCTCTCCACCAACCATG |
| Non-symbiotic hemoglobin 2 gene | LOC_Os03g12510 | TGCTTGAGACGATCAAGGAG | ACGAGCACAATGCAACAAAG |
| Actin gene | LOC_Os03g50885 | TTGCTGACAGGATGAGCAAG | TGGAATGTGCTGAGAGATGC |
| Beta-tubulin gene | LOC_Os01g59150 | GCTGACCACACCTAGCTTTGG | AGGGAACCTTAGGCAGCATGT |
