# Supplementary material for: Characterization of contrasting rice (Oryza sativa L.) genotypes reveals the Pi-efficient schema for phosphate starvation tolerance
Source: BMC Plant Biol. 2021 Jun 21;21:282. doi: 10.1186/s12870-021-03015-4 (PMC8215752; doi:10.1186/s12870-021-03015-4)
Supplement: Supplementary file 4 — Additional file 4: Supplementary Methods. Detailed method for the experiments performed. [file 12870_2021_3015_MOESM4_ESM.doc]

**Characterization of contrasting rice (*Oryza sativa* L.) genotypes reveals the Pi-efficient schema for phosphate starvation tolerance**

**Suresh Kumar, Pallavi, Chetna Chugh, Karishma Seem, Santosh Kumar, K. K. Vinod, and Trilochan Mohapatra**

**Supplementary Methods**

**Plant Materials, Growth Conditions and P-starvation Stress Imposition**

Contrasting rice genotypes (Pusa-44, P-deficiency sensitive and NIL-23, P-deficiency stress tolerant), were used in the study. Pusa-44, a high-yielding, long-duration (150 days) variety, is sensitive to P-deficiency stress. The Near Isogenic Line 23 (NIL-23, a BC_3_F_6_ progeny) used in the present study is one of the selected NILs developed through backcrossing between IR64-*Pup1*-F (containing *Pup1* QTL from Kasalath) crossed with Pusa-44 (Supplementary Fig. S20). The plants of BC_3_F_6_ generation used in the present study had recovered >95% of the recurrent parent genome. The individuals of NIL population developed through backcross breeding were screened under field conditions by growing them in P-deficient and P-sufficient conditions. Some of the better performing NILs for agronomic traits under P-deficiency stress were selected (data not shown), and the best performer (NIL-23) among these (Supplementary Fig. S21) screened out based on the biochemical (total acid phosphatase activity) and physiological (root-shoot biomass ratio) parameters at vegetative stage (data not shown) was used for further detailed morphological, biochemical, physiological and molecular analyses.

Mature seeds of the contrasting rice genotype [Pusa-44, recurrent parent, and NIL-23, along with the original *Pup1* QTL donor (Kasalath) used specifically for certain comparative evaluation] were surface-sterilized with 3% sodium hypochlorite solution for 5 min, followed by washing three times with sterilized double-distilled water. Seeds were soaked overnight in sterile distilled water, the imbibed seeds were spread on 5 layers of wet germination paper sheets placed in 140 mm Petri plates, and incubated in dark for 2 days at 37 °C for germination. The germinated seeds with coleoptiles and coleorhiza were rolled in moist germination paper sheets, placed on a layer of butter paper (resembling ‘cigar roll’) to facilitate vertical/upward growth of shoot and downward growth of roots for ease in transfer of the seedlings in hydroponic culture. The cigar rolls were placed in a jam bottle filled with the hydroponic medium (Supplementary Fig. S22), and incubated in natural light at 30 °C day/22 °C night, 80−85% humidity in a glasshouse. Ten-days-old rice seedlings were transferred to hydroponic culture in PusaRicH medium [1]. The PusaRicH medium was prepared (using RO water) with or without H_3_PO_4_ (inorganic phosphorus), and pH of the medium was maintained at 5.2. A set of plants were grown in the medium containing sufficient (16 ppm Pi), deficient (1 or 4 ppm Pi), or no (0 ppm Pi) phosphorus. Throughout the experiment, pH of the medium was maintained at 5.2 using 1.0 N KOH, and the medium was replaced at 3 days interval. Morphological data were recorded on the 45-day-old (vegetative/tillering stage) plants. Shoots and roots were collected from 45-day-old plants and the tissue samples were used for various biochemical, physiological and molecular analyses.

**Estimation of Acid Phosphatase Activity**

Acid phosphatase (APase) activity was estimated following the protocol described by Johnson et al. [2]. Fresh shoot and root tissues (100 mg) was homogenized in 5 ml chilled citrate buffer (0.1 M, pH 5.2), and the mixture was centrifuged at 7800x g for 15 min at 4 ℃. The supernatant was used as enzyme extract, and in 0.1 ml of the enzyme extract 0.4 ml of chilled citrate buffer (0.1 M, pH 5.2) and 0.5 ml ρ-nitrophenol (10 mM ρ-NP, pH 5.2) were added. The reaction mixture was incubated at 22 ℃ for 10 min, the reaction was stopped by adding 2.0 ml of Na_2_CO_3_ (0.2 M), and absorbance was recorded at 405 nm using spectrophotometer. APase activity (μM) was calculated using standard curve of ρ-NP concentration (10-100 µM).

To estimate the secreted acid phosphatases, from roots of the 45-day-old plants, roots of the plant were kept submerged in 100 ml (10 mM) ρ-NPP in a glass bottle and incubated at 30 °C for 60 min. Then, 100 ml of 0.25 M NaOH was added to stop the reaction. Absorbance was recorded at 412 nm using spectrophotometer, and the activity in μM was calculated using ρNP standard curve following the procedure described by Tian et al. [3].

To assess an effect of the exudates from roots of the plants on pH of the medium (initially adjusted at 5.2) used for hydroponics, pH of the culture medium was measured on alternate day starting from 42 days till 48 days of the age of the plants.

**Estimation Root-Shoot Biomass Ratio**

Roots and shoots from the 45-day-old plants of the contrasting rice genotypes grown under control (full or 16 ppm Pi) and P-starvation stress were collected. Fresh weight of the plant tissues was recorded immediately. The tissues were loosely wrapped in aluminum foil, dried at 42 °C in electric hot-air oven for 4−5 days until they achieved constant weight, and the dry biomass was recorded. The root-shoot biomass ratio was calculated using the formula:

Root dry biomass

Root-Shoot biomass ratio= -------------------------

Shoot dry biomass.

**Estimation of Chlorophyll Content**

Total chlorophyll content was estimated in fresh leaf tissues (0.5 g) using dimethyl sulfoxide (DMSO) method as described by Hiscox and Israelstam [4]. The leaf was cut into small (5 mm) pieces using scalpel blade and transferred into a test tube. The tissues were submerged in 2 ml DMSO and incubated at 60 °C for 20 min. The chlorophyll thus extracted was collected in a fresh tube by decanting. Another 2 ml of DMSO was added to the leaf tissues, and incubated again at 60 °C for 20 min. The extracts were pooled together, and the final volume was made up to 10 ml with DMSO. Absorbance of the chlorophyll extract was recorded at 645 and 663 nm against DMSO as blank using a spectrophotometer. Total chlorophyll content was calculated on fresh weight (FW) basis using the following formula:

Total chlorophyll content (mg/g FW) = (20.2 × A_645_) + (8.02 × A_663_).

**Estimation of Phosphorus Content**

Total phosphorus content in shoot and root of the plants was determined using the Vanadate-molybdate method as described below [5]. The plant tissues were dried at 60 °C until reached to a constant weight. A fixed amount (1.0 g) of the dried tissue sample was double-acid digested with 10 ml of HNO_3_ and 3.5 ml of HClO_4_ by heating at 200 °C for 90 min. The extract was then filtered using Whatman filter paper No.1, and the volume was made up to 100 ml with double distilled water. 25 ml of the extract was taken in a 50 ml volumetric flask, 10 ml of Vanadate-molybdate solution was added, and the final volume was made up to 50 ml with double distilled water. The content was mixed properly, incubated for 10 min for color development, and absorbance was recorded at 420 nm. Standard curve was prepared by taking varying concentration of phosphorus (1.0, 1.5, 2.0, 2.5, 3.0, 4.0, and 5.0 mg), color development, taking OD at 420 nm, and plotting P concentration on X axis and OD at Y axis. P content in the sample (%) was calculated using the standard curve.

**RNA Isolation, cDNA Library Preparation and Illumina Sequencing**

To investigate the effects of P-starvation in contrasting rice (Pusa-44, P-deficiency sensitive; NIL-23, P-deficiency tolerant) genotypes, transcriptome analysis was performed for shoots and roots tissues. Towards this end, whole transcriptome data from shoot and root tissues from 45-day-old rice plants grown hydroponically in P-sufficient (16 ppm Pi) or –starved (0 ppm Pi) PusaRicH medium was analyzed. The samples were submerged in RNAlater, stored at 4 °C for 24 h, and then at −20 °C till further processing. Total RNA was isolated from the plant tissues using Trizol method with 3 biological replications. About 250 mg tissue was ground into fine powder using liquid nitrogen, and the powdered tissue was transferred into DEPC-treated 1.5 ml Eppendorf tube containing 1.0 ml of TRIzol reagent. The contents were mixed vigorously for 1 min and then incubated at 25 °C for 5 min. Chloroform (200 µl) was then added, mixed, and incubated at 25 °C for 5 min. The content was then centrifuged at 12000 rpm for 15 min. The upper aqueous phase was carefully removed (avoiding the intake of interface) and transferred to an RNase-free 1.5 ml Eppendorf tube, an equal volume of isopropanol was added and mixed well by slowly inverting the tubes. The tunes were incubated overnight at −80 °C. The tubes were then centrifuged at 14000 rpm for 30 min, and the pellet was washed with 1.0 ml of 75% ethanol by centrifugation at 12000 rpm for 15 min. The pellet was then air-dried for 5 min, and the RNA pellet was dissolved in 50 μl of DEPC-treated water.

The total RNAs, thus isolated, was treated with DNase using DNase I Kit (Thermo Scientific, Cat. # E0523) following the manufacturer’s protocol. The treated total RNAs pellet was air-dried and dissolved in 50 µl of DEPC-treated water. To assess the quantity, purity and integrity of RNAs, OD value was recorded at 260 and 280 nm (for assessment based on A260/A280 ratio) was observed using Nanodrop (Thermo Scientific), and denaturing agarose gel electrophoresis. For RNA quality/integrity check, agarose gel electrophoresis was performed using DEPC-treated electrophoresis tank and other accessories. A 1.2% denaturing agarose gel was prepared by dissolving 0.6 g of agarose in 43.5 ml of DEPC-treated water, heated to dissolve agarose, cooled down to 60 °C, and then 5 ml of 10× MOPS buffer, 1.5 ml of 37% formaldehyde, 2.5 µl of ethidium bromide solution (from a stock solution of 10 mg/ml) were added and missed well avoiding the introduction of air bubble. The content was then poured into the prepared casting tray placed with comb, and allowed to polymerize by leaving for 60 min at room temperature.

RNAs were denatured in sterile RNase-free Eppendorf tube by mixing the sample with an equal volume of 2× RNA-loading dye (Thermo Scientific, Cat. # R0641), mixed well, heated at 65 °C for 10 minutes, and then placed immediately in ice for 10 min. 2 µl of the total RNAs sample was loaded in the well of the gel, and run at 50 V for 30 min, and then the gel was visualized and photographed using a gel imaging system (GeneSnap, SYNGENE). Equal amount of total RNAs from each of the 3 biological replicates were pooled together, used to prepare cDNAs for quantitative PCR analysis.

**Quality Check, RNA-Seq Data Analysis, and Mapping to Rice Genome**

For RNA-seq analysis, equal amount of total RNAs pooled from each of the 3 biological replicates were used to prepare a total of 16 libraries for 2 the rice genotypes (Pusa-44 − P-deficiency sensitive, NIL-23 − P-deficiency tolerant), 2 tissues (root and shoot), 2 different concentration (16 ppm − control, and 0 ppm − stress treatment) of phosphorus used to grow plants, and 2 technical replications. The quality and integrity of total RNAs were checked by using Qubit RNA Assay Kit and Qubit 4 (Invitrogen, USA), and a Bioanalyzer system (Agilent Technologies, USA). The mRNA enrichment, RNA fragmentation, the first and second strand cDNA synthesis and purifying, sequencing adaptors ligation and PCR amplification were performed as per TruSeq RNA Sample Preparation kit (Illumina). Transcriptome libraries were generated by standard illumina protocol and sequenced on Hiseq 2500 using 2× 150 bp paired-end chemistry. Raw sequence data were submitted to NCBI Sequence Read Archive (SRA) database under the BioProject ID PRJNA667189.

FastQC 0.11.7 (http://www.bioinformatics.babraham.ac.uk/projects/fastqc) analysis was used to assess the quality of raw RNA reads for all the 16 libraries. After removing the low-quality reads, mismatches, and adaptor sequences from the raw reads, clean reads were generated [6]. The cleaned RNA reads were mapped on to the rice reference genome (Rice Genome Annotation Project (RGAP) database, [http://rice.plantbiology.msu.edu](http://rice.plantbiology.msu.edu/)) reference genome using the HiSat2 2.1.0 aligner [7] with default parameters and assembly were performed with Stringtie package. The read counts were generated from the alignment files using Feature Counts software (Subread package 1.6.2) with default parameters [8], based on rice RAP-DB annotation gtf file version 1.0.38. The number of mapped clean reads for each gene was counted and normalized into the reads per kilo base per million value (RPKM) values. Differentially expressed genes (DEGs) were analyzed by using Deseqq2 package to compare between treatment conditions. The log_2_ fold change (log_2_-FC) of expression profiles (as RPKM) was calculated between the P-starvation treated and unstressed (control) for each genotype. A false discovery rate (FDR) ≤0.1, *p* <0.05 and log_2_FC ≥±1 was used as the threshold to judge the significance of difference.

**Gene Ontology Analysis**

To perform gene ontology (GO) enrichment analysis for the differentially expressed genes AgriGO v2 software was used (http://bioinfo.cau.edu.cn/agriGO). The analysis identifies enriched GO terms by comparing a query list of gene identifiers and their corresponding GO terms, with a background population list from which the query list was derived. The background list of genes and GO annotations was extracted from Rice Genome Annotation Project (RGAP) database. Sets of the most important, differentially expressed, candidate genes were identified based on the information available for the P-deficiency tolerance-specific genes.

**Validation of Differentially Expressed Genes by RT-qPCR**

To confirm the RNA-Seq results, seven DEGs (four up-regulated and three down-regulated) were selected randomly for quantitative (RT-qPCR) analysis following the MIQE guidelines. The cDNA was synthesized from mRNA using 2 μg of total RNAs sample following the Manufacturer’s instruction (Invitrogen Superscript III First Strand Synthesis Kit, Cat. # 18080051). The synthesized cDNA was diluted five-times and used as a template for RT-qPCR validation of the randomly selected differentially expressed genes (DEGs) using an Agilent Real-Time PCR system with KAPA SYBR fast mix. RT-qPCR was performed in 10 µl reaction mix, and thermal cycler was programmed for an initial denaturation at 95 °C for 3 min, followed by 40 cycles each of 5 sec denaturation at 94 °C, 20 sec annealing at 60 °C and 40 sec extension at 72 °C. Amplification data collection was set at the end of each extension step. The data was analyzed through melt curve analysis to check the specificity of PCR amplification. The relative gene expression was determined by the 2^-ΔΔCT^ method. *Actin* (LOC_Os03g50885) was used as house-keeping/internal control gene. All the primers used for RT-qPCR validation of genes are listed in Supplemental Table S12.

**References**

1. Sharma S, Borah P, Meena MK, Bindraban P, Pandey R. Evaluation of genotypic variation for growth of rice seedlings under optimized hydroponics medium. Indian J Genet. 2018;78:292−301.

2. Johnson CB, Holloway BR, Smith H, Grierson D. Isoenzymes of acid phosphatase in germinating peas. Planta. 1973;115:1–10.

3. Tian J, Wang C, Zhang Q, He X, Whelan J, Shou H. Over expression of *OsPAP10*, a root –associated acid phosphatase, increased extracellular organic phosphorus utilization in rice. J Integr Plant Biol. 2012;54:631–639.

4. Hiscox JD, Israelstam GF. Different methods of chlorophyll extraction. Canad J Bot. 1979;57:1332–1332. doi:10.1139/b79−163

5. Hanson WC. The photometric determination of phosphorus in fertilizers using the phosphovanado- molybdate complex. J Sci Food Agric. 1950;1:172–173.

6. Bolger AM, Lohse M, Usadel B. Trimmomatic: A flexible trimmer for Illumina sequence data. Bioinformatics. 2014;30:2114–2120. doi:10.1093/bioinformatics/btu170.122

7. Pertea M, Kim D, Pertea GM, Leek JT, Salzberg SL. Transcript-level expression analysis of RNA-seq experiments with HISAT, StringTie and Ballgown. Nat Protoc. 2016;11:1650–1667. doi:10.1038/nprot.2016.095

8. Liao Y, Smyth GK, Shi W. An efficient general-purpose program for assigning sequence reads to genomic features. Bioinformatics. 2014;30:923–930. doi:10.1093/bioinformatics/btt656
