# Supplementary material for: Characterization of contrasting rice (Oryza sativa L.) genotypes reveals the Pi-efficient schema for phosphate starvation tolerance
Source: BMC Plant Biol. 2021 Jun 21;21:282. doi: 10.1186/s12870-021-03015-4 (PMC8215752; doi:10.1186/s12870-021-03015-4)
Supplement: Supplementary file 5 — Additional files 5: Supplementary Figures S1 to S22. [file 12870_2021_3015_MOESM5_ESM.zip › Supplementary Figure S1.pdf]

Characterization of contrasting rice (*Oryza sativa* L.) genotypes reveals the Pi-efficient schema for phosphate starvation tolerance

Suresh Kumar, Pallavi, Chetna Chugh, Karishma Seem, Santosh Kumar, K. K. Vinod, and Trilochan Mohapatra

Supplementary Figure S1: Effect of P-starvation on growth and development of rice seedlings (20-day-old) grown hydroponically in cigar rolls. Pusa-44 grown in PusaRicH medium with 16 ppm Pi (A), Pusa-44 grown in PusaRicH medium with 0 ppm Pi (B), NIL-23 grown in PusaRicH medium with 16 ppm Pi (C), NIL-23 grown in PusaRicH medium with 0 ppm Pi (D), Kasalath grown in PusaRicH medium with 16 ppm Pi (E), Kasalath grown in PusaRicH medium with 0 ppm Pi (F).

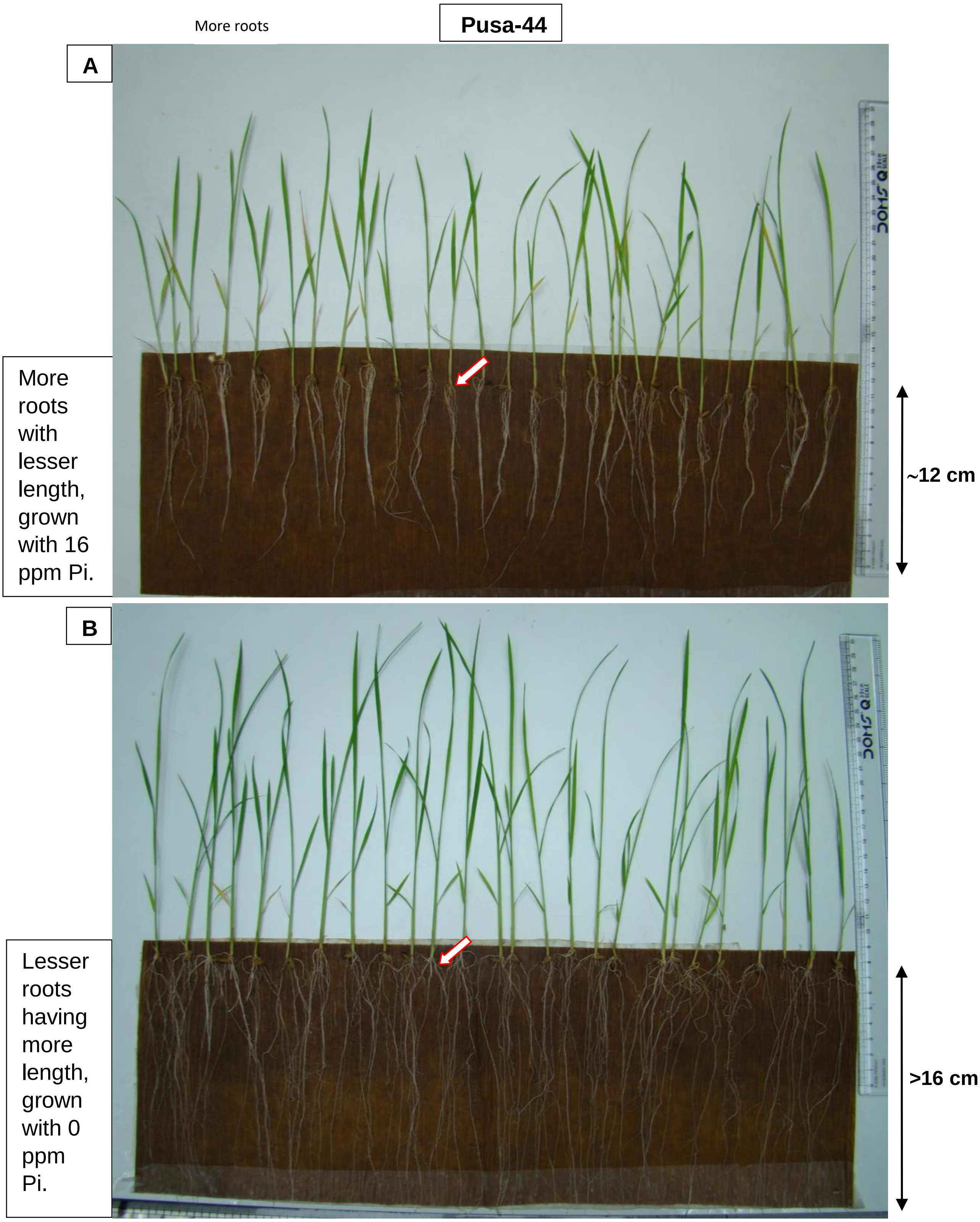

NIL-23

C

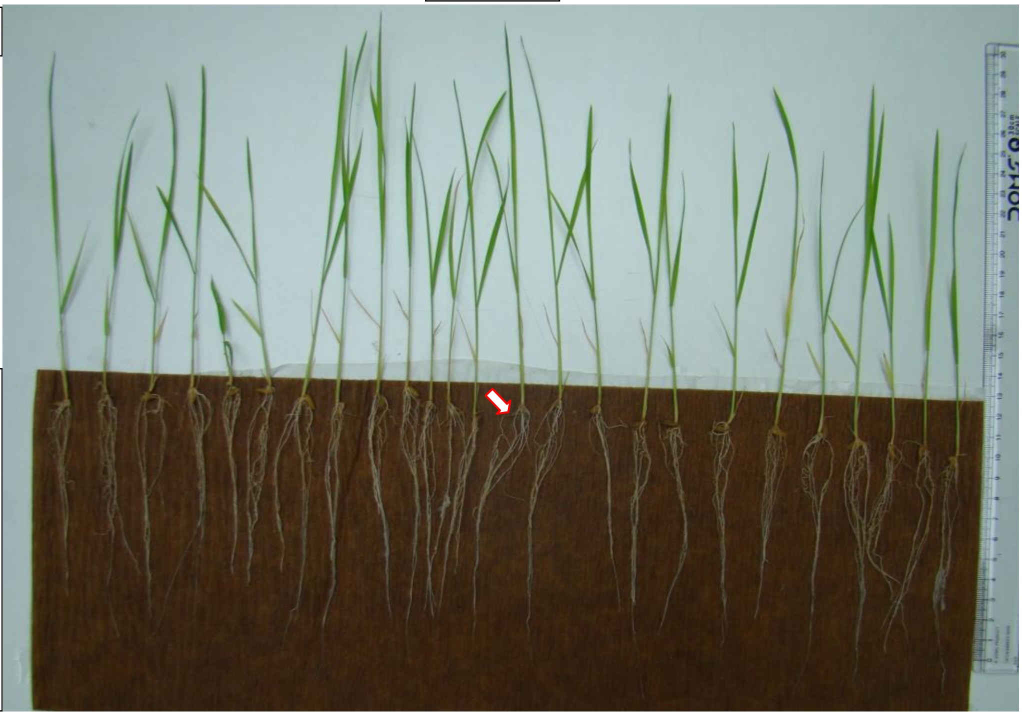

More roots with lesser length, grown with 16 ppm Pi.

~12 cm

D

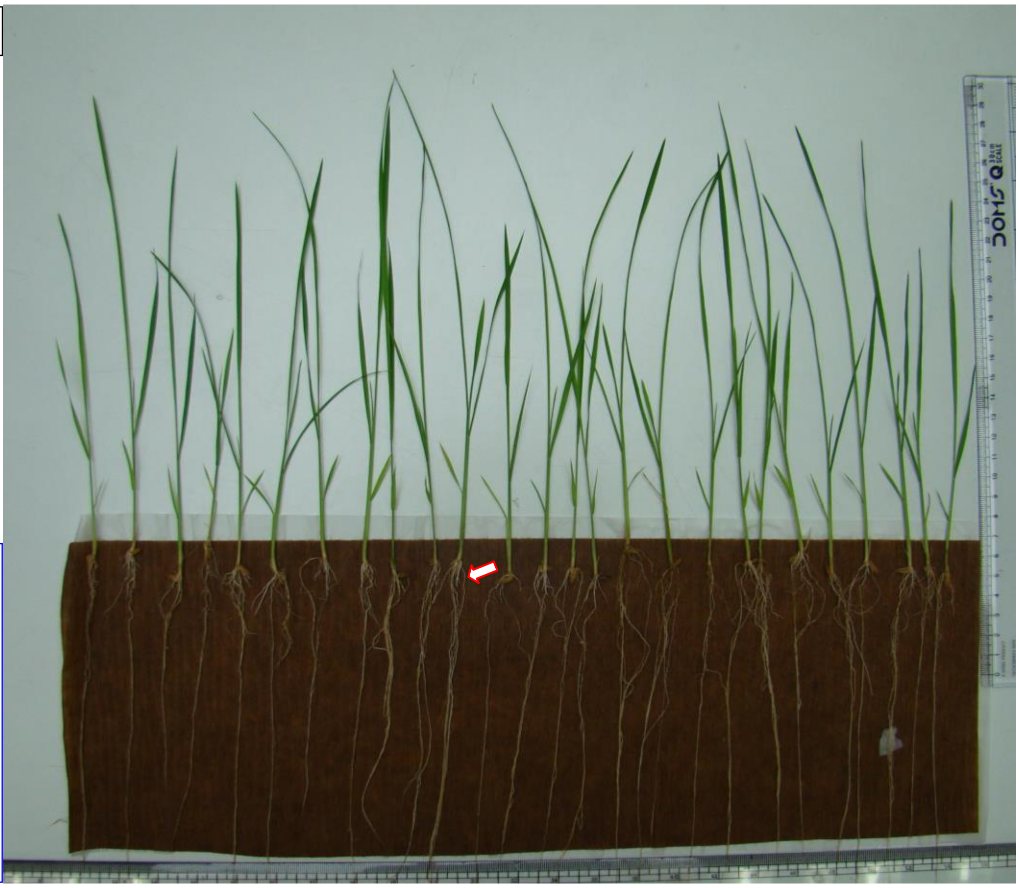

Lesser roots with more length, grown with 0 ppm Pi.

>18 cm

Kasalath

E

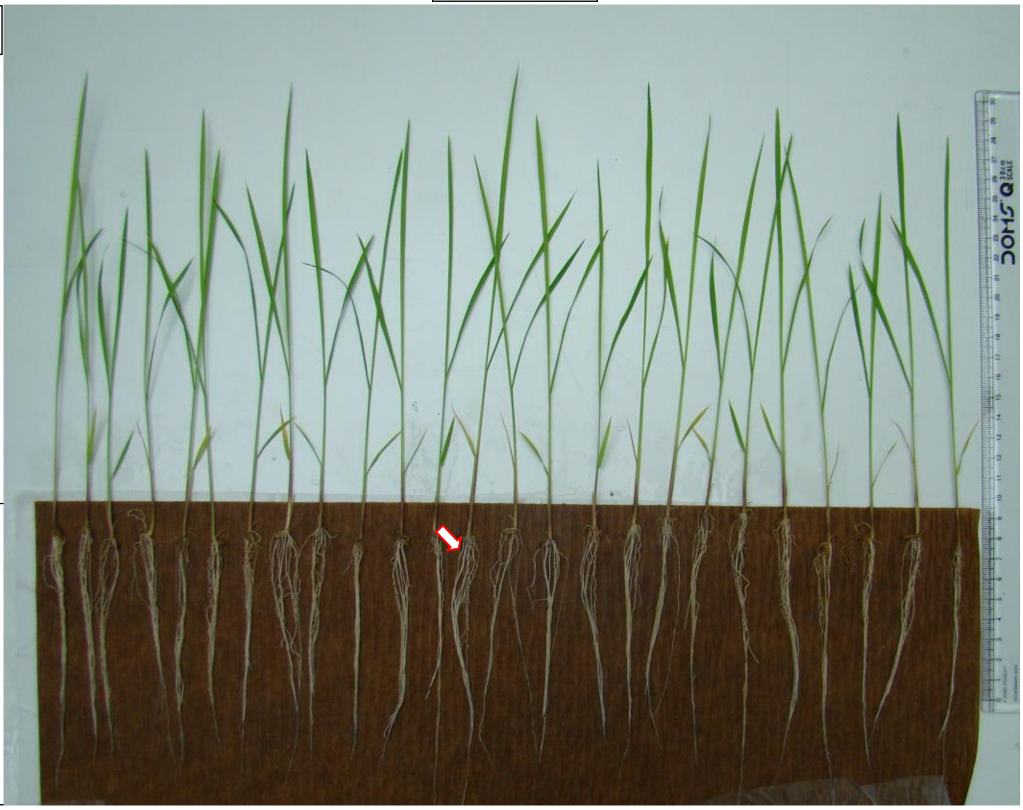

More roots with lesser length, grown with 16 ppm Pi.

~12 cm

F

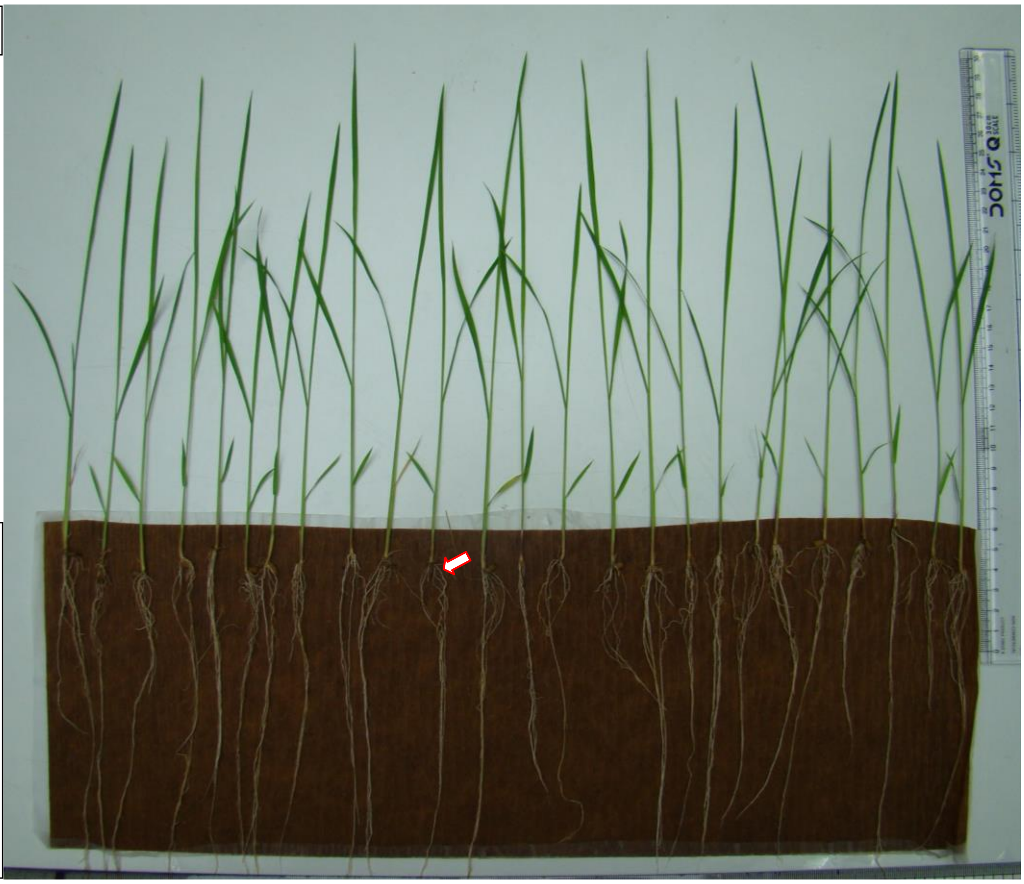

Lesser roots with more length, grown with 0 ppm Pi.

>19 cm
