# Supplementary material for: Characterization of contrasting rice (Oryza sativa L.) genotypes reveals the Pi-efficient schema for phosphate starvation tolerance
Source: BMC Plant Biol. 2021 Jun 21;21:282. doi: 10.1186/s12870-021-03015-4 (PMC8215752; doi:10.1186/s12870-021-03015-4)
Supplement: Supplementary file 1 — Additional file 1: Supplementary Tables S1 to S2. Comparative analysis of root-shoot biomass ratio in the contrasting rice genotypes along with the donor parent, Effect of the root-secreted acid phosphatase on pH of the hydroponic medium. [file 12870_2021_3015_MOESM1_ESM.zip › 12870_2021_3015_MOESM1_ESM/Supplementary Table S1.doc]

**Characterization of contrasting rice (*Oryza sativa* L.) genotypes reveals the Pi-efficient schema for phosphate starvation tolerance**

**Suresh Kumar, Pallavi, Chetna Chugh, Karishma Seem, Santosh Kumar, K. K. Vinod, and Trilochan Mohapatra**

**Supplementary** **Table S1** | Comparative analysis of root-shoot biomass ratio in the contrasting rice genotypes along with the donor parent.

| **Genotype** | **Role/Characteristics of the plant** | **Root biomass (mg)^*^** | |  | **Shoot biomass (mg)^*^** | |  | **Root−Shoot biomass ratio** | |
| --- | --- | --- | --- | --- | --- | --- | --- | --- | --- |
|  |  | **16 ppm Pi** | **0 ppm Pi** |  | **16 ppm Pi** | **0 ppm Pi** |  | **16 ppm Pi** | **0 ppm Pi** |
| Pusa-44 | Recurrent parent, P-deficiency sensitive | 200±12 | 100±9 |  | 950±24 | 350±16 |  | 0.210 | 0.286 |
| NIL-23 | Near isogenic line, P-deficiency tolerant | 290±14 | 185±11 |  | 980±25 | 370±17 |  | 0.296 | 0.500 |
| Kasalath | Donor parent, P-deficiency tolerant | 294±13 | 174±12 |  | 995±24 | 375±18 |  | 0.295 | 0.464 |

The plants were grown hydroponically in the PusaRicH medium containing 16 ppm (Control) or 0 ppm Pi (Treated) for 45 days. **^*^**The value represents mean of three biological and three technical replications ±SD.
