# Supplementary material for: Characterization of contrasting rice (Oryza sativa L.) genotypes reveals the Pi-efficient schema for phosphate starvation tolerance
Source: BMC Plant Biol. 2021 Jun 21;21:282. doi: 10.1186/s12870-021-03015-4 (PMC8215752; doi:10.1186/s12870-021-03015-4)
Supplement: Supplementary file 1 — Additional file 1: Supplementary Tables S1 to S2. Comparative analysis of root-shoot biomass ratio in the contrasting rice genotypes along with the donor parent, Effect of the root-secreted acid phosphatase on pH of the hydroponic medium. [file 12870_2021_3015_MOESM1_ESM.zip › 12870_2021_3015_MOESM1_ESM/Supplementary Table S2.doc]

**Characterization of contrasting rice (*Oryza sativa* L.) genotypes reveals the Pi-efficient schema for phosphate starvation tolerance**

**Suresh Kumar, Pallavi, Chetna Chugh, Karishma Seem, Santosh Kumar, K. K. Vinod, and Trilochan Mohapatra**

**Supplementary Table S2** | Effect of the root-secreted acid phosphatase on pH of the hydroponic medium.

| **Genotype** | **Age of the plant**  **Phosphorus**  **concentration** | **Decrease in pH of the medium at tillering stage of plant growth**  **(5.2 − pH recorded after 2 days)** | | | | |
| --- | --- | --- | --- | --- | --- | --- |
|  |  | **42 day** | **44 day** | **46 day** | **48 day** | **Mean** |
| **Pusa-44** | 0 ppm | 0.41 | 0.46 | 0.49 | 0.39 | 0.44 |
|  | 1 ppm | 1.59 | 1.35 | 1.37 | 1.57 | 1.47 |
|  | 4 ppm | 1.59 | 1.62 | 1.63 | 1.60 | 1.61 |
|  | 16 ppm | 1.78 | 1.89 | 1.55 | 1.65 | 1.72 |
| **NIL-23** | 0 ppm | 0.11 | 0.02 | 0.11 | 0.08 | 0.08 |
|  | 1 ppm | 1.39 | 1.41 | 1.46 | 1.38 | 1.41 |
|  | 4 ppm | 1.55 | 1.68 | 1.56 | 1.69 | 1.62 |
|  | 16 ppm | 1.85 | 1.94 | 1.67 | 1.81 | 1.82 |
| **Kasalath** | 0 ppm | 0.01 | 0.01 | 0.01 | 0.02 | 0.01 |
|  | 1 ppm | 1.49 | 1.55 | 1.59 | 1.53 | 1.54 |
|  | 4 ppm | 1.77 | 1.35 | 1.79 | 1.89 | 1.70 |
|  | 16 ppm | 1.85 | 1.78 | 1.81 | 1.75 | 1.80 |

The plants were grown hydroponically in the PusaRicH medium containing different concentration [16 ppm (Control), 4 ppm, 1 ppm (P-deficiency), or 0 ppm (P-starvation)] of Pi. The pH of medium was initially adjusted at 5.2, and change in pH was checked after every 2 days starting from 40 days of the age of plants. Pusa-44 (P-deficiency sensitive), NIL-23 and Kasalath (*Pup1* QTL donor), P-deficiency tolerant genotypes, were used in study. The value for change in pH represents mean of three replications.
