# Supplementary material for: Characterization of contrasting rice (Oryza sativa L.) genotypes reveals the Pi-efficient schema for phosphate starvation tolerance
Source: BMC Plant Biol. 2021 Jun 21;21:282. doi: 10.1186/s12870-021-03015-4 (PMC8215752; doi:10.1186/s12870-021-03015-4)
Supplement: Supplementary file 5 — Additional files 5: Supplementary Figures S1 to S22. [file 12870_2021_3015_MOESM5_ESM.zip › Supplementary Figure S11.doc]

**Characterization of contrasting rice (*Oryza sativa* L.) genotypes reveals the Pi-efficient schema for phosphate starvation tolerance**

**Suresh Kumar, Pallavi, Chetna Chugh, Karishma Seem, Santosh Kumar, K. K. Vinod, and Trilochan Mohapatra**

**Supplementary Figure S11:** Gene ontology (GO) analysis of molecular functions under P-starvation stress in roots of the contrasting rice genotypes. Under-represented GO terms in stress-sensitive Pusa-44 (**A**), and under-represented GO terms in stress-tolerant NIL-23 rice genotype (**B**).
