# Supplementary material for: Characterization of contrasting rice (Oryza sativa L.) genotypes reveals the Pi-efficient schema for phosphate starvation tolerance
Source: BMC Plant Biol. 2021 Jun 21;21:282. doi: 10.1186/s12870-021-03015-4 (PMC8215752; doi:10.1186/s12870-021-03015-4)
Supplement: Supplementary file 5 — Additional files 5: Supplementary Figures S1 to S22. [file 12870_2021_3015_MOESM5_ESM.zip › Supplementary Figure S2.doc]

**Characterization of contrasting rice (*Oryza sativa* L.) genotypes reveals the Pi-efficient schema for phosphate starvation tolerance**

**Suresh Kumar, Pallavi, Chetna Chugh, Karishma Seem, Santosh Kumar, K. K. Vinod, and Trilochan Mohapatra**

**Supplementary Figure S2:** Effect of different phosphorus concentration (0 to 40 ppm) on growth and development of Pusa-44 (45-day-old) rice plants grown in PusaRicH medium.

**0 ppm Pi**

**4 ppm Pi**

**8 ppm Pi**

**12 ppm Pi**

**16 ppm Pi**

**20 ppm Pi**

**24 ppm Pi**

**28 ppm Pi**

**32 ppm Pi**

**36 ppm Pi**

**40 ppm Pi**
