# Supplementary material for: Characterization of contrasting rice (Oryza sativa L.) genotypes reveals the Pi-efficient schema for phosphate starvation tolerance
Source: BMC Plant Biol. 2021 Jun 21;21:282. doi: 10.1186/s12870-021-03015-4 (PMC8215752; doi:10.1186/s12870-021-03015-4)
Supplement: Supplementary file 5 — Additional files 5: Supplementary Figures S1 to S22. [file 12870_2021_3015_MOESM5_ESM.zip › Supplementary Figure S20.doc]

**Characterization of contrasting rice (*Oryza sativa* L.) genotypes reveals the Pi-efficient schema for phosphate starvation tolerance**

**Suresh Kumar, Pallavi, Chetna Chugh, Karishma Seem, Santosh Kumar, K. K. Vinod, and Trilochan Mohapatra**

**Supplementary Figure S20:** Plants of the rice genotypes used in the present study. Plants of an aus landrace Kasalath at (i) vegetative and (ii) reproductive stage (A), NIL-23 in seed setting stage (B), and Pusa-44 (C).

**A**

**Kasalath**

**Kasalath**

**(i)**

**Vegetative**

**(ii)**

**Reproductive**

**Pusa-44**

**NIL-23**

**C**

**B**

**Reproductive**

**Reproductive**
