# Supplementary material for: Characterization of contrasting rice (Oryza sativa L.) genotypes reveals the Pi-efficient schema for phosphate starvation tolerance
Source: BMC Plant Biol. 2021 Jun 21;21:282. doi: 10.1186/s12870-021-03015-4 (PMC8215752; doi:10.1186/s12870-021-03015-4)
Supplement: Supplementary file 5 — Additional files 5: Supplementary Figures S1 to S22. [file 12870_2021_3015_MOESM5_ESM.zip › Supplementary Figure S3.doc]

**Characterization of contrasting rice (*Oryza sativa* L.) genotypes reveals the Pi-efficient schema for phosphate starvation tolerance**

**Suresh Kumar, Pallavi, Chetna Chugh, Karishma Seem, Santosh Kumar, K. K. Vinod, and Trilochan Mohapatra**

**Supplementary Figure S3:** Effect of P-starvation on growth and development of rice at vegetative stage (45-day-old). Pusa-44 grown in PusaRicH medium containing 0 ppm (treatment) or 16 ppm (control) Pi (A), NIL-23 grown in PusaRicH medium containing 0 ppm or 16 ppm Pi (B), Kasalath grown in PusaRicH medium containing 0 ppm or 16 ppm of Pi (C).

**A**

**0 ppm Pi**

**16 ppm Pi**

**B**

**C**

**0 ppm Pi**

**16 ppm Pi**

**0 ppm Pi**

**16 ppm Pi**
