# Supplementary material for: Characterization of contrasting rice (Oryza sativa L.) genotypes reveals the Pi-efficient schema for phosphate starvation tolerance
Source: BMC Plant Biol. 2021 Jun 21;21:282. doi: 10.1186/s12870-021-03015-4 (PMC8215752; doi:10.1186/s12870-021-03015-4)
Supplement: Supplementary file 5 — Additional files 5: Supplementary Figures S1 to S22. [file 12870_2021_3015_MOESM5_ESM.zip › Supplementary Figure S4.doc]

**Characterization of contrasting rice (*Oryza sativa* L.) genotypes reveals the Pi-efficient schema for phosphate starvation tolerance**

**Suresh Kumar, Pallavi, Chetna Chugh, Karishma Seem, Santosh Kumar, K. K. Vinod, and Trilochan Mohapatra**

**Supplementary Figure S4:** Effect of P-starvation on shoot growth and development in rice (45-day-old) plants. Pusa-44 grown in PusaRicH medium containing different (0 ppm, 1 ppm, 4 ppm or 16 ppm) concentration of Pi (A), NIL-23 grown in PusaRicH medium containing different concentration of Pi (B), Kasalath grown in PusaRicH medium containing different concentration of Pi (C).

**0 ppm Pi**

**1 ppm Pi**

**4 ppm Pi**

**16 ppm Pi**

**71% reduction**

**in height** (compared to the control, # Aiv)

**(i)**

**(ii)**

**(iii)**

**(iv)**

**(i)**

**Pusa-44**

**NIL-23**

**Kasalath**

**A**

**B**

**C**

**(ii)**

**(iii)**

**(iv)**

**0 ppm Pi**

**1 ppm Pi**

**4 ppm Pi**

**15 ppm Pi**

**47% reduction**

**in height** (compared to the control, # Biv)

**(ii)**

**(i)**

**(iii)**

**(iv)**

**51% reduction**

**in height** (compared to the control, # Civ)
