# Supplementary material for: Characterization of contrasting rice (Oryza sativa L.) genotypes reveals the Pi-efficient schema for phosphate starvation tolerance
Source: BMC Plant Biol. 2021 Jun 21;21:282. doi: 10.1186/s12870-021-03015-4 (PMC8215752; doi:10.1186/s12870-021-03015-4)
Supplement: Supplementary file 5 — Additional files 5: Supplementary Figures S1 to S22. [file 12870_2021_3015_MOESM5_ESM.zip › Supplementary Figure S5.doc]

**Characterization of contrasting rice (*Oryza sativa* L.) genotypes reveals the Pi-efficient schema for phosphate starvation tolerance**

**Suresh Kumar, Pallavi, Chetna Chugh, Karishma Seem, Santosh Kumar, K. K. Vinod, and Trilochan Mohapatra**

**Supplementary Figure S5:** Effect of P-starvation on growth and development of leaf at vegetative (45-day-old) stage of the rice plants.

**44% reduction**

**in length, 73% reduction in leaf area.**

**41% reduction**

**in length, 70% reduction in leaf area.**

**51% reduction**

**in length,**

**81% reduction in leaf area.**

Pusa-44

NIL-23

Kasalath

Kasalath

NIL-23

Pusa-44

**16 ppm Pi Pusa-44**

**0 ppm Pi Pusa-44**
