# Supplementary material for: Characterization of contrasting rice (Oryza sativa L.) genotypes reveals the Pi-efficient schema for phosphate starvation tolerance
Source: BMC Plant Biol. 2021 Jun 21;21:282. doi: 10.1186/s12870-021-03015-4 (PMC8215752; doi:10.1186/s12870-021-03015-4)
Supplement: Supplementary file 5 — Additional files 5: Supplementary Figures S1 to S22. [file 12870_2021_3015_MOESM5_ESM.zip › Supplementary Figure S9.doc]

**Characterization of contrasting rice (*Oryza sativa* L.) genotypes reveals the Pi-efficient schema for phosphate starvation tolerance**

**Suresh Kumar, Pallavi, Chetna Chugh, Karishma Seem, Santosh Kumar, K. K. Vinod, and Trilochan Mohapatra**

**Supplementary Figure S9:** Phosphorus content (%) in plant tissues on dry weight (DW) basis in the 45-day-old plants of rice genotypes. Pusa-44 (**A**), NIL-23 (**B**), and Kasalath (**C**) grown hydroponically under control (16 ppm inorganic phosphorus), P-deficiency (4 or 1 ppm Pi), or P-starvation (0 ppm Pi) stress.
